# Sweet-taste liking is associated with preference for less risky and immediate rewards in economic decision-making

**DOI:** 10.3389/fpsyg.2026.1764306

**Published:** 2026-05-20

**Authors:** Anna Davidovich, Anna N. Shestakova, Nina Arzumanyan, Ksenia Panidi

**Affiliations:** 1 Centre for Cognition and Decision Making, Institute for Cognitive Neuroscience, HSE University, Moscow, Russia; 2 International Laboratory of Social Neurobiology, Institute for Cognitive Neuroscience, HSE University, Moscow, Russia

**Keywords:** impulsive choice, intertemporal choice, reward sensitivity, risk aversion, sweet-taste liking

## Abstract

**Background:**

Delay discounting refers to the tendency to choose sooner, smaller rewards over larger, later rewards. Many previous studies link this tendency positively to reward sensitivity, yet the specific mechanisms behind this association remain poorly understood. Reward sensitivity may relate to delay discounting through at least three possible pathways: increased sensitivity to reward size, increased sensitivity to reward immediacy, or increased sensitivity to risk. In this study, we use a biologically grounded measure of reward sensitivity, reflected by hedonic preference for sweet taste, to identify which of these mechanisms may be involved in the relationship between reward sensitivity and delay discounting.

**Methods:**

In the first experiment (*N* = 49), participants performed a standardized Kampov-Polevoy sweet-taste liking test, a lottery choice task, and a Balloon Analogue Risk Task (BART). Reward sensitivity was measured using the Kampov-Polevoy sweet-taste liking test, which categorizes participants into sweet-likers and sweet-dislikers based on their liking ratings of sucrose solutions. Regression analysis was used to examine whether the sweet-liking phenotype was significantly associated with risk-taking propensity. In the second experiment (*N* = 100), participants completed the same sweet-liking test, and the lottery choice task as in Experiment 1, along with the delay discounting task to evaluate impulsivity. Regression analysis was used to analyze the association between the sweet-liking phenotype and delay discounting and to test whether this relationship is explained by differences in the sensitivity to reward size, reward immediacy, or risk preferences between the sweet-liking phenotypes.

**Results:**

In Experiment 1, sweet-likers were more risk-aversive compared to sweet-dislikers in a lottery choice task, although no such effect was observed in the BART task. The same tendency toward risk aversion was observed in Experiment 2. In the delay discounting task, sweet-liking participants showed greater preference for sooner smaller reward which was associated with increased sensitivity to the reward immediacy, and not reward size. However, this relationship was partly explained by the greater risk aversion of the sweet-likers compared to sweet-dislikers.

**Conclusion:**

Our results suggest that the positive relationship between reward sensitivity and impulsivity may be explained by a stronger preference for certain rewards, rather than a heightened desire for immediate rewards.

## Introduction

Impulsivity is a complex trait that includes a broad range of behaviors. It is generally defined as a tendency to act quickly without sufficient thought, often leading to decisions that carry potential risks or negative outcomes (Evenden, 1999). Because impulsivity includes multiple dimensions, it is usually assessed using different methodological approaches (Mitchell and Potenza, 2014). One widely used measure is the delay discounting task, which captures the preference for smaller, immediate rewards over larger delayed ones. Many studies suggest that delay discounting can be considered a behavioral marker of impulsivity in healthy participants, as well as in various disorders. In particular, this measure of impulsivity has been shown to be correlated with substance abuse in alcohol (MacKillop et al., 2010; Mitchell et al., 2005) and nicotine overconsumption (Johnson et al., 2007), as well as a range of behavioral addictions (Weinsztok et al., 2021).

However, the neurophysiological mechanisms of delay discounting remain poorly understood. A growing body of research suggests that reward sensitivity may play an important role in impulsive behavior in general, and in delay discounting in particular (Zhao et al., 2025). Reward sensitivity is a psychological trait in which an individual is more likely to be motivated by the prospect of receiving tangible rewards. This heightened response to rewards may result in a stronger inclination to engage in behaviors that are likely to yield such rewards (Costumero et al., 2013). It is typically assessed in different ways, including personality questionnaires and behavioral tasks. Self-report measures include the Behavioral Inhibition System/Behavioral Activation System (BIS/BAS) scales (Carver and White, 1994) and the Sensitivity to Punishment and Sensitivity to Reward Questionnaire (SPSRQ) (Torrubia et al., 2001). The standardized Kampov-Polevoy sweet-taste liking test (Kampov-Polevoy et al., 1997), which evaluates hedonic responses to sweet taste (Damiano et al., 2014), can be considered as a physiological approach to measuring reward sensitivity. Based on the results of this test individuals can be categorized into two major phenotypes—the sweet-liking (SL) and the sweet-disliking (SDL) phenotype (Garbutt et al., 2009; Kampov-Polevoy et al., 1997, 2006; Kranzler et al., 2001).

Although reward sensitivity has often been viewed as a unitary construct, its universality has been questioned in recent literature (Stephens et al., 2010). Growing evidence suggests that sensitivity to reward is domain-specific: individuals may show heightened responsiveness to one type of reward (e.g., food or sweet taste) but not to others (e.g., music or social rewards) (Mas-Herrero et al., 2026; Christoffersen, 2025). At the neural level, sensitivity to different reward types appears to depend on partially distinct brain circuits. For instance, even within the same brain region (mPFC), non-overlapping neuronal ensembles represent social versus non-social (sucrose) rewards (Isaac et al., 2024).

Some evidence suggests that sweet-taste liking might serve as a biological indicator of reward sensitivity. For example, sweet-liking has been linked to preference for more rewarding foods (Han et al., 2020). In addition, a recent meta-analysis of fMRI studies on sweet taste reported tentative evidence for reward-related caudate activity during sweeter taste stimulation (Roberts et al., 2020). However, the authors emphasized that this finding was driven by a single study by Eiler et al. (2018), and the meta-analysis did not identify reliable orbitofrontal cortex activation related to sweetener tasting, although this area is linked to the reward processing and value-based decision making (O’Doherty, 2004). The SL phenotype is associated with increased risks of substance use disorder development, and some studies argue that sweet-liking reflects a genetic or neurobiological vulnerability overlapping with addiction pathways (van Amsterdam and van den Brink, 2025; Lange et al., 2010; Kampov-Polevoy et al., 2003). Importantly, altered reward processing has been indicated as an important risk factor for the development of addictions (Baskin-Sommers and Foti, 2015; Luijten et al., 2017).

Moreover, the SL phenotype has been shown to represent a relatively stable and heritable trait. Twin studies indicate that approximately half of the variation in this phenotype is genetically determined (Keskitalo et al., 2007; Mennella et al., 2005), and it remains stable throughout life (Bae and Kang, 2024; Thompson et al., 1976). Taken together, this evidence suggests that although sweet-taste liking may not capture any universal reward sensitivity, it may still represent a biological measure of sensitivity to a primary sensory reward, which might be correlated with other aspects of value-based decision-making.

Previous studies indicate that the SL phenotype may be associated with impulsivity and delay discounting. Clinically, the SL phenotype is a significant risk factor for SUDs, which are also commonly associated with increased delay discounting. This is evidenced by its association with a 2.5-fold increase in the odds of SL occurrence in individuals with a family history of alcoholism (Kampov-Polevoy et al., 2003) and its broader link to various SUDs (van Amsterdam and van den Brink, 2025). Research has identified the SL phenotype and childhood sugar consumption as predictive markers for subsequent SUD development (Mehlig et al., 2018). Animal models demonstrate that the rewarding effects of sweeteners can rival or exceed those of cocaine (Ahmed et al., 2013; Lenoir et al., 2007) and that excessive sugar consumption can lead to addictive-like behaviors, providing a neurobiological parallel to human SUD etiology.

Importantly, heightened impulsivity is widely considered a core behavioral feature of SUDs (Perry and Carroll, 2008; de Wit, 2009). Individuals with substance dependence frequently exhibit difficulties in delaying gratification and demonstrate a pronounced preference for immediate rewards, even when such choices entail long-term negative consequences. Delay discounting paradigms consistently reveal steeper discounting rates in individuals with alcohol, nicotine, and other substance dependencies compared to healthy controls (MacKillop et al., 2011; Amlung et al., 2017), suggesting that a stronger preference for immediate rewards reflects increased vulnerability to addictive behaviors. Finally, a study in humans demonstrated that participants with the SL phenotype exhibited steeper delay discounting (Weafer et al., 2014), while participants showing higher preference for sweet taste in wine were characterized by increased impulsivity scores (Saliba et al., 2009).

However, previous studies did not explore the specific pathways through which reward sensitivity might be linked to delay discounting. At least three different mechanisms can be hypothesized.

First, reward sensitivity might be associated with the propensity to take risks. In the context of delay discounting this association may be particularly important since the immediate option always involves minimal to no risk, while the delayed option is always associated with some degree of uncertainty. Hence, a highly risk averse individual may choose an immediate smaller reward to avoid risk, and will thus appear more impulsive in a delay discounting task (Lopez-Guzman et al., 2018). But the relationship between the two phenomena may be deeper than a mere distortion of measurement. For example, discounting is observed to be steeper for certain outcomes than for the uncertain ones, while risk aversion is usually lower for future risks than for imminent risks (Epper and Fehr-Duda, 2024). Some studies suggest considering the decrease of value due to a time delay or due to risks as a common process of ‘discounting’, referring to the two phenomena as delay and probability discounting, respectively (Escobar et al., 2023). Experimental measures have shown that a hyperbolic function provides a better fit to both measures and indicates a moderate correlation between the two (Escobar et al., 2023). At the neural level, delay discounting and risk preferences have been observed in partly overlapping regions (Luhmann et al., 2008; Peters and Büchel, 2009). Both delay discounting and risky decision making engage a common valuation network, including the ventromedial prefrontal cortex (vmPFC), ventral striatum, and posterior cingulate cortex (PCC). These regions are known to encode the subjective value of different choice options, regardless of whether decisions involve time delays or uncertainty (Kable and Glimcher, 2007; Bartra et al., 2013). These findings have been interpreted within different theoretical frameworks. Early dual-system models suggested that intertemporal choices reflect the interaction between two partially distinct neural systems (McClure et al., 2007). A reward-related system, involving limbic and striatal regions such as the ventral striatum and medial orbitofrontal cortex, responds strongly to immediately available rewards. In contrast, a control system associated with lateral prefrontal regions [particularly dorsolateral prefrontal cortex (dlPFC)] is thought to support deliberation and self-control, promoting choices of delayed rewards. Experimental evidence using transcranial magnetic stimulation has shown that disruption of left lateral prefrontal cortex (lPFC) function increases impulsive choices, further supporting its role in self-control during intertemporal decision-making (Figner et al., 2010). However, a more recent approach suggests that a single valuation system performs value computation (Kable and Glimcher, 2010, 2007). This approach highlights that prefrontal regions are not considered as a separate control system, but rather part of the same valuation network involved in computing subjective value. Importantly, although delay discounting and risk preferences share common neural pathways, they do not always move in the same direction behaviorally. For example, at the developmental level, adolescents have been shown to have a steeper delay discounting than adults, while at the same time showing the highest degree of risk seeking compared to all other age groups (Rosenbaum and Hartley, 2019). This divergence becomes particularly evident in populations characterized by altered reward processing and valuation mechanisms. For example, pathological gamblers are typically characterized by steeper delay discounting of rewards compared to healthy controls, while at the same time showing increased tolerance to risk (Miedl et al., 2012; Petry, 2001; Weinsztok et al., 2021).

Second, reward sensitivity may manifest as a heightened sensitivity to reward magnitude. Individuals with higher reward sensitivity exhibit greater motivation to pursue rewards, as documented in both behavioral and neuroimaging studies (van Duijvenvoorde et al., 2016, 2014). Within a delay discounting framework, this heightened sensitivity to reward size can exert competing influences. An increase in the delayed reward’s magnitude may promote the choice of the delayed, larger option. Conversely, an increase in the immediate reward’s magnitude may disproportionately amplify the attractiveness of the immediate choice.

Finally, reward sensitivity may be linked to the modulation of subjective reward value in relation to time delay. Immediacy of reward might induce a stronger reaction in individuals with high reward sensitivity since immediate rewards might be considered as more tangible or salient. At the neural level, immediate rewards activate the brain areas that are associated with incentive salience and reward processing (such as the ventral striatum), similar to sugar-related reward sensitivity (Daniel and Pollmann, 2014; von Molitor et al., 2021). The delayed rewards by contrast require the recruitment of brain regions involved in perspective-taking and future-oriented processing, particularly the right temporoparietal junction (rTPJ). Activation of this region may contribute to perceiving delayed rewards as more psychologically distant than immediate ones (Soutschek et al., 2020; O'Connell et al., 2018).

The present study aimed to explore which of the three components—risk preferences, sensitivity to reward size, or sensitivity to reward immediacy—might mediate the link between reward sensitivity and delay discounting. We report the results of two experiments. Across both experiments, we found that the SL phenotype is primarily associated with risk aversion. In Experiment 1, using a binary lottery choice task, SL individuals demonstrated a significantly higher propensity to avoid risks, independent of reward size or probability. This result was replicated in Experiment 2 on a separate sample, which showed that SL participants were more likely to choose a safe option, particularly under high-risk conditions. In a delay discounting task, the SL phenotype was initially associated with the sensitivity to reward immediacy, and was not associated with the delay length or the size of reward. However, this relationship was partially mediated by risk aversion, becoming only marginally significant after controlling for the general propensity to avoid risk. Collectively, our results suggest that the link between reward sensitivity and delay discounting may be specifically explained by a heightened preference for certain rewards.

## Experiment 1

### Materials and methods

#### Participants

Forty-nine healthy participants (33 females, mean age = 24 years, SD = 3.93) completed the experiment. Participants were recruited using the university’s database and online advertisements. Eligibility criteria included being between 18 and 40 years of age, not being pregnant, and having no diabetes or any psychiatric or neurological disorders. Exclusion criteria included adherence to specific dietary regimens, undergoing therapeutic treatments, smoking more than five cigarettes per week (including electronic cigarettes), and having a background in mathematics, physics, economics, finance, or computer science. We excluded participants with advanced education in these disciplines to avoid potential bias, since their understanding of statistical and probabilistic concepts could affect performance on tasks designed to assess naturalistic decision-making. All experimental procedures followed the university ethical committee’s guidelines and regulations (HSE University IRB approval 7.02.2022, 80(2)). Participants provided written informed consent prior to the beginning of the experiment. They were compensated with 250 monetary units in local currency (~8.5 USD by purchasing power parity at the time of the experiment).

#### Experimental procedure

Participants attended a 1.5-h experimental session. They completed a series of behavioral tasks and self-report measures, which were administered in a randomized order to minimize order effects. Experiment 1 included three behavioral tasks: the sweet-taste liking test, the balloon analogue risk task (BART), and the lottery choice task. In the sweet-taste liking test, participants rated their preference for water solutions with varying concentrations of sucrose. The BART was used to measure risk-taking behavior. Participants were instructed to inflate a virtual balloon to accumulate points, with the risk of losing them if the balloon burst. The lottery choice task was also used to measure risk preferences. Participants made choices between monetary lotteries that differed in reward magnitude and probability. All tasks were coded and implemented in PsychoPy (version 2022.1.4).

The order of the tasks was randomized between participants as follows. Twenty-four randomly selected participants first completed the sweet-taste liking test, followed by two decision-making tasks: the lottery choice task and BART. The remaining 25 participants completed the decision-making tasks first, and then performed the sweet-taste liking test. The order of the risky choice tasks was fixed: all participants first completed the lottery choice task, followed by BART. This order was chosen to avoid potential bias in lottery responses that could be induced by successes and failures in the BART task.

Following the behavioral tasks, participants completed a set of questionnaires including the Sensation Seeking (SS) and the Novelty Seeking (NS) scales. These measures were used in Experiment 1 because previous research has shown that the Sensation Seeking scale is a strong predictor of risky behavior (Horvath and Zuckerman, 1993), and that higher scores on the Novelty Seeking scale have been positively associated with risk preference (Wang et al., 2015).

To control for potential effects of hunger on task performance, participants were instructed to abstain from eating and from consuming beverages such as coffee or tea for at least 1.5 h prior to the session.

#### Measures

##### Sweet-taste liking task

Sweet-liking preferences were assessed using the methodology described by Kampov-Polevoy et al. (1997, 2003, 2001). Participants rated five concentrations of different sucrose solutions (0.05, 0.10, 0.21, 0.42, and 0.83 M sucrose) across five randomized blocks (25 trials total). Before starting the main trials, participants were given a practice sample of water to ensure they understood the procedure. For each trial, they received 2 mL of the solution administered through a syringe, swished it for 5 s, and then spat it out into a separate container. Participants then rinsed their mouths with still water before evaluating each sample. They rated sweetness intensity and liking on 200-mm visual analog scales: 0 to 200 for sweetness (0 = not sweet, 200 = very sweet) and −100 to +100 for liking (−100 = strongly dislike, +100 = strongly like). Participants who rated the 0.83 M solution as their most pleasurable sample were classified as sweet-likers. Participants who rated one of the lower sucrose concentrations (0.05, 0.10, 0.21, or 0.42 M) as the most pleasant were categorized as sweet-dislikers (Kampov-Polevoy et al., 1997, 2003, 2001).

##### Balloon analogue risk task

Risk-taking behavior was measured using the BART paradigm (Lejuez et al., 2002). In this task, participants made repeated decisions about inflating a virtual balloon displayed on a computer screen. Each pump increased the potential monetary reward by 0.3 RUB but also increased the risk of the balloon exploding. If the balloon exploded, the accumulated earnings for that trial were lost. Participants could choose to stop inflating at any time and press the “Collect money” button which would transfer the accumulated sum to a permanent bank. Each participant completed 30 balloon trials. The explosion point was randomly determined for each balloon and was not known to the participants, thereby introducing uncertainty into the task. The average number of adjusted pumps, i.e., the average number of pumps for the balloons that did not burst was used as a measure of individual risk-taking tendency (Lejuez et al., 2002). Higher numbers of adjusted pumps were interpreted as a greater willingness to take risks. At the end of the experiment, the amount of money that participants managed to deposit in their permanent bank during the BART task was added to their participation fee. The experimental instructions clearly informed the participants that the monetary reward accumulated in the BART task would be paid out in real currency.

##### Lottery choice task

**Figure 1:**
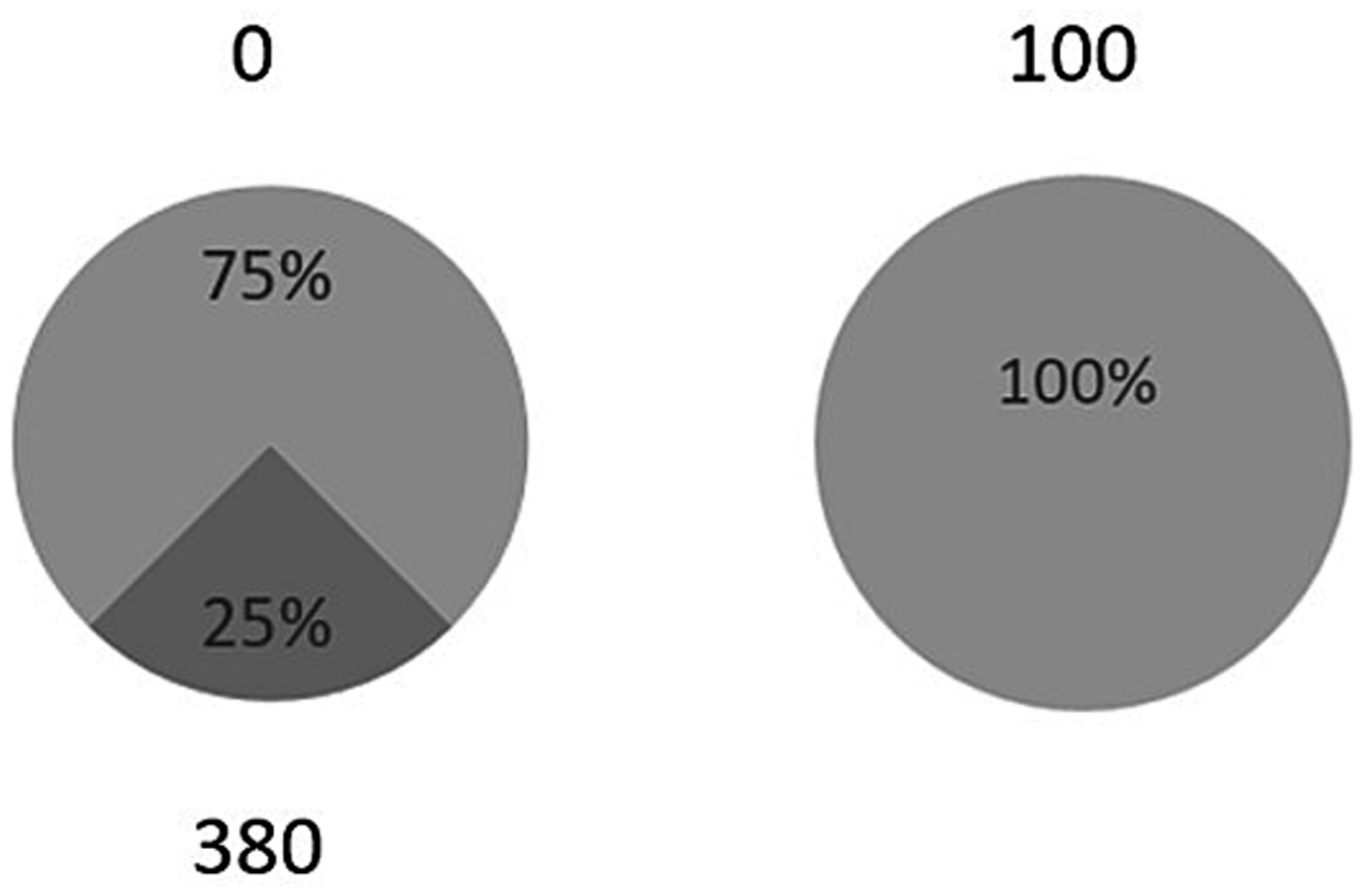
An example of a trial in the lottery choice task. A participant is offered a choice between a risky lottery (left option), where he/she can win 380 monetary units (MU) with a 25% probability or receive 0 MU with a 75% probability, and a certain option (right option), where he/she receives 100 MU for sure.

Risk preferences were assessed using the lottery choice task, following the methodology employed in previous studies (Holt and Laury, 2002; Lopez-Guzman et al., 2018). This task measured participants’ decision-making under risk by offering a choice between a smaller guaranteed reward and a larger, probabilistic one. The task included 60 trials. In each trial, participants chose between two options: a certain reward [100 monetary units (MU)] and a risky lottery offering a higher reward (ranging from 100 to 500 MU in increments of 20) with a probability of 25, 50%, or 75%. The positions of the sure and risky options on the screen (left or right) were randomized across trials. An example trial is shown in Figure 1.

At the end of the experiment, one trial out of all 60 trials was randomly selected, and participants were paid based on the option they chose in this randomly selected trial, in addition to their participation fee. The experimental instructions informed participants that this procedure would be used to determine their final reimbursement for the experiment.

#### Data analysis

To evaluate whether the perceived sweetness and the subjective liking of the sucrose solutions differed between the sweet-likers and sweet-dislikers across sucrose concentrations, we first assessed normality within each group. Normality was evaluated using the Shapiro–Wilk test. Since the assumption of normality did not hold for several sucrose concentrations, the Wilcoxon rank-sum test was used for all sweetness and liking comparisons. Since the assumptions of normality held for the SS and the NS, Welch’s t-test was used to assess differences between the SL and SDL groups on these measures.

To test whether the sweet-liking status was linked to risky behavior, regression analysis was conducted. In the lottery choice task, to evaluate the relationship between sweet-liking status and risky behavior, two models were estimated. Each model was a logistic model with subject-level random effects. The dependent variable was a binary variable equal to 1 if a participant chose a risky option in a particular trial, and equal to zero otherwise. The main independent variable of interest was the sweet-liking status (equal to 1 for sweet-likers and 0 for sweet-dislikers). Additional variables included lottery characteristics—the reward amount of the risky option and the winning probability, modeled as a categorical variable with levels 25 percent, 50 percent, and 75 percent. Participants’ age and gender and the order of the tasks were included as control variables. The first model did not include any interaction terms and had the following structure (Equation 1.1):


$$\begin{array}{l} \mathrm{Pr}{\left(\text{choose risky option}\right)}_{\mathrm{ij}}=\beta_{0}+\beta_{1}\text{Sweet likin}g_{i}+\beta_{2}\text{Task orde}r_{i}+\beta_{3}\text{Risky rewar}d_{\mathrm{ij}}+\beta_{4}{\left(\text{Probability of risky reward}=0.5\right)}_{\mathrm{ij}}+\\ \;\;\;\;\;\;\;\;\;\;\;\;\;\;\;\;\;\;\;\;\;\;\;\;\;\;\;\;\;\;\;\;\;\;\;\;\;\;\;\;\;\;\;\;\;\;\;\;\;\;\;\;\;\;\;\;\;\;\;\;\;\;\beta_{5}{\left(\text{Probability of risky reward}=0.75\right)}_{\mathrm{ij}}+\text{γControl}s_{i}+\varepsilon_{\mathrm{ij}} \end{array}$$ (1.1)

The second model had the same structure but additionally included the interaction terms of the sweet-liking status with the lottery reward and winning probability (Equation 1.2):


$$\begin{array}{l} \mathrm{Pr}{\left(\text{choose risky option}\right)}_{\mathrm{ij}}=\beta_{0}+\beta_{1}\text{Sweet likin}g_{i}+\beta_{2}\text{Task orde}r_{i}+\\ \;\;\;\;\;\;\;\;\;\;\;\;\;\;\;\;\;\;\;\;\;\;\;\;\;\;\;\;\;\;\;\;\;\;\;\;\;\;\;\;\;\;\;\;\;\;\;\;\;\;\;\;\;\;\;\;\;\;\;\;\;\;\;\beta_{3}\text{Risky rewar}d_{\mathrm{ij}}+\beta_{4}{\left(\text{Probability of risky reward}=0.5\right)}_{\mathrm{ij}}+\\ \;\;\;\;\;\;\;\;\;\;\;\;\;\;\;\;\;\;\;\;\;\;\;\;\;\;\;\;\;\;\;\;\;\;\;\;\;\;\;\;\;\;\;\;\;\;\;\;\;\;\;\;\;\;\;\;\;\;\;\;\;\;\;\beta_{5}{\left(\text{Probability of risky reward}=0.75\right)}_{\mathrm{ij}}+\\ \;\;\;\;\;\;\;\;\;\;\;\;\;\;\;\;\;\;\;\;\;\;\;\;\;\;\;\;\;\;\;\;\;\;\;\;\;\;\;\;\;\;\;\;\;\;\;\;\;\;\;\;\;\;\;\;\;\;\;\;\;\;\;\beta_{6}\text{Sweet likin}g_{i}\times \text{Risky rewar}d_{\mathrm{ij}}+\\ \;\;\;\;\;\;\;\;\;\;\;\;\;\;\;\;\;\;\;\;\;\;\;\;\;\;\;\;\;\;\;\;\;\;\;\;\;\;\;\;\;\;\;\;\;\;\;\;\;\;\;\;\;\;\;\;\;\;\;\;\;\;\beta_{7}\text{Sweet likin}g_{i}\times {\left(\text{Probability of risky reward}=0.5\right)}_{\mathrm{ij}}+\\ \;\;\;\;\;\;\;\;\;\;\;\;\;\;\;\;\;\;\;\;\;\;\;\;\;\;\;\;\;\;\;\;\;\;\;\;\;\;\;\;\;\;\;\;\;\;\;\;\;\;\;\;\;\;\;\;\;\;\;\;\;\beta_{8}\text{Sweet likin}g_{i}\times {\left(\text{Probability of risky reward}=0.75\right)}_{\mathrm{ij}}+\\ \;\;\;\;\;\;\;\;\;\;\;\;\;\;\;\;\;\;\;\;\;\;\;\;\;\;\;\;\;\;\;\;\;\;\;\;\;\;\;\;\;\;\;\;\;\;\;\;\;\;\;\;\;\;\;\;\;\;\;\;\;\gamma \cdot \text{Control}s_{i}+\varepsilon_{\mathrm{ij}} \end{array}$$ (1.2)

The model without the interaction terms (Model 1.1) allowed to evaluate whether sweet-likers generally prefer more risky or less risky options, while including the interaction terms (Model 1. 2) allowed to assess whether sweet-liking moderates the effects of various option characteristics (reward amount and winning probability) on choice.

In the BART, to assess the link between sweet-liking preferences and risky behavior we used the adjusted number of pumps as a dependent variable. This measure captures the average number of pumps on trials where the balloon did not explode. The model is as follows (Equation 1.3):


$$\text{Adjusted number of pump}s_{i}=\beta_{0}+\beta_{1}\text{Sweet likin}g_{i}+\beta_{2}\text{Task orde}r_{i}+\gamma \cdot \text{Control}s_{i}+\varepsilon_{i}$$ (1.3)

Analysis was conducted in R (version 4.2.2; R Core Team, 2024). We used the lme4 package for fitting fit mixed-effects logistic regressions.

### Results

#### Sweet-taste liking test

Participants were classified as sweet-likers or sweet-dislikers based on their liking ratings for the highest sucrose concentration (0.83 M). Twelve participants were classified as sweet-likers (3 males and 9 females); the remaining thirty-seven (13 males and 24 females) were classified as sweet-dislikers.

**Figure 2:**
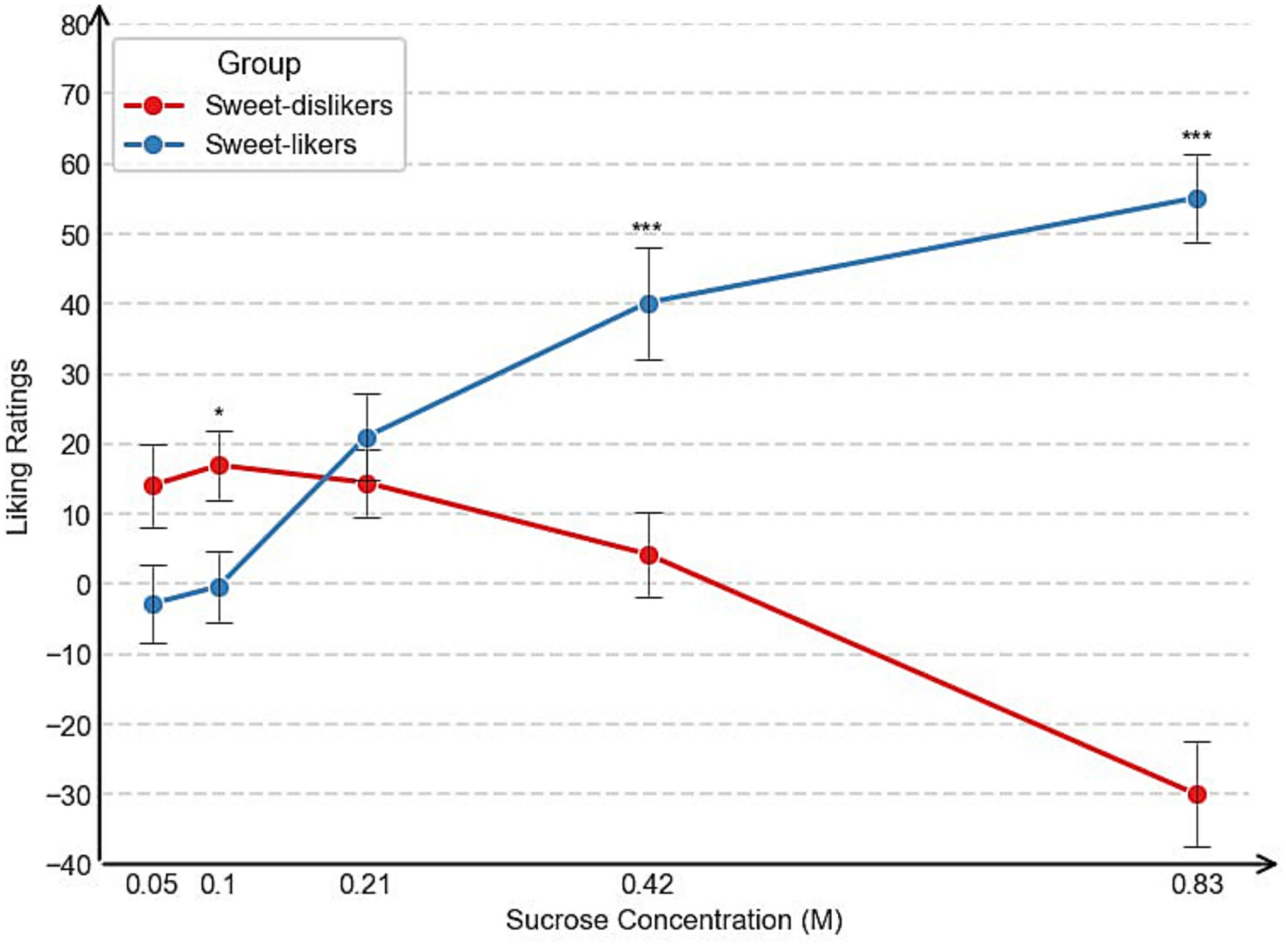
Mean liking across concentrations between sweet-liking groups, Experiment 1. ***p < 0.001, **p < 0.01, *p < 0.05.

Table 1 summarizes the group means for perceived sweetness and liking across five sucrose concentrations (0.05, 0.10, 0.21, 0.42, and 0.83 M). Sweetness ratings did not significantly differ between the groups at any concentration (all *p* > 0.16), indicating that group differences in liking were not driven by differences in perceived sweetness. However, sweet-likers gave significantly lower liking ratings for the 0.10 M sucrose concentration, but significantly higher ratings for the higher concentrations, compared to sweet-dislikers. This pattern is clearly illustrated in Figure 2. More detailed information on the distribution of responses to the sweet-liking task across sweet-liking groups can be found in Supplementary material.

**Table 1:** Mean sweetness and liking ratings across concentrations between sweet-liking groups, Experiment 1.

		Concentration				
		0.05	0.10	0.21	0.42	0.83
Mean sweetness	Sweet-dislikers	23.08	43.41	84.16	128.22	161.51
	Sweet-likers	30.67	37.5	76.5	126	149.17
	*p*-value	0.93	0.25	0.33	0.90	0.17
Mean liking	Sweet-dislikers	14.05	16.97	14.49	4.27	−30.05
	Sweet-likers	−2.83	−0.33	21	40.17	55.17
	*p*-value	0.07	0.02	0.88	0.001	0.00

#### Personality scales

Scores on the Sensation Seeking and Novelty Seeking scales did not significantly differ between sweet-likers and sweet-dislikers (*p* = 0.64; *p* = 0.33 respectively). Detailed analyses of these measures are reported in Supplementary material.

#### Lottery choice task

**Figure 3:**
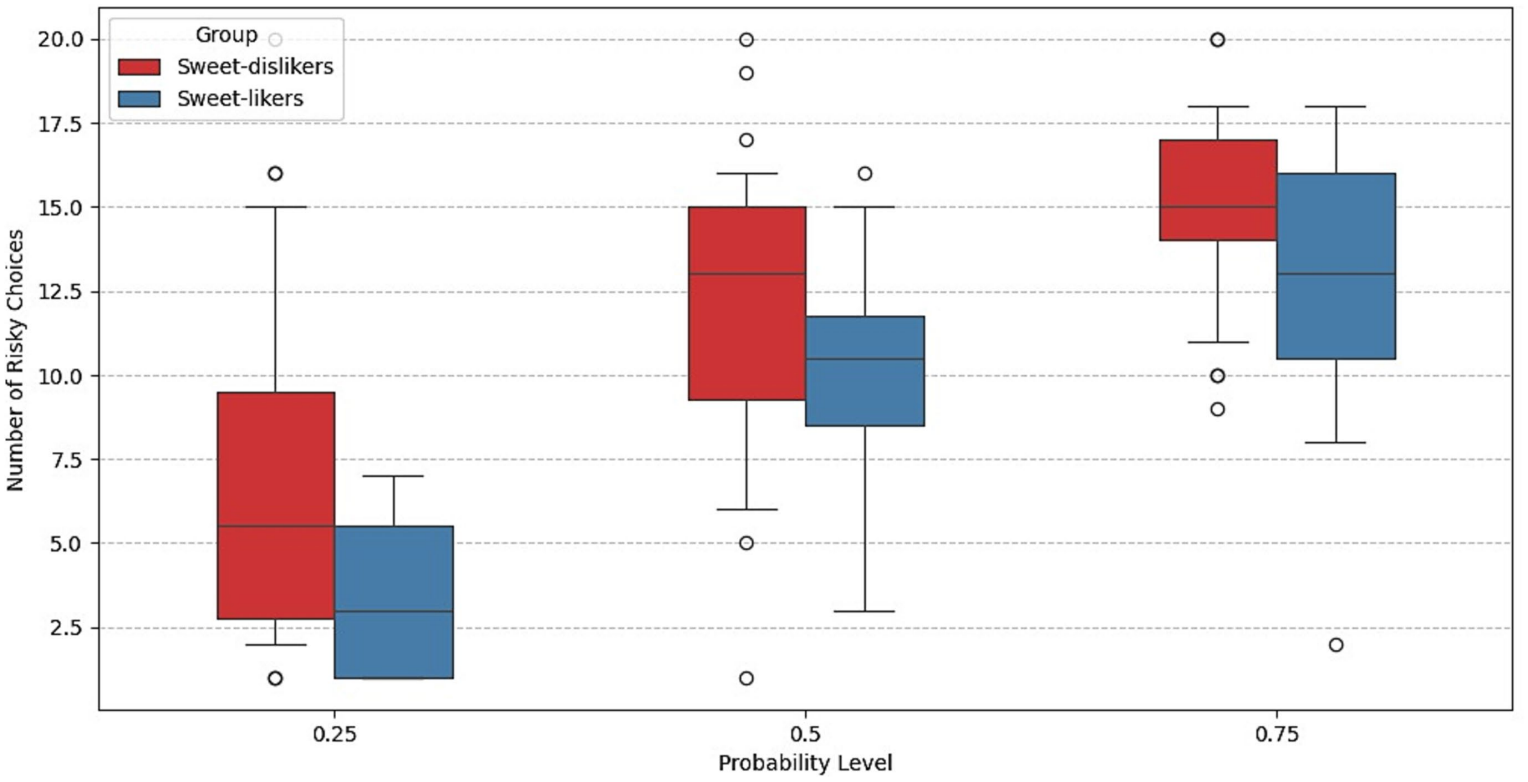
Number of risky choices in the lottery choice task across trials with different probabilities in Experiment 1.

Figure 3 indicates that the number of risky choices increases with the probability of obtaining the risky option in both groups. More detailed information on the distribution of responses to the lottery choice task across sweet-liking groups can be found in Supplementary material. To assess group differences in risk behavior more precisely, we next employed regression analysis using a logit model with subject-level random effects.

Table 2 presents the estimation results for two logistic regression models with subject-level random effects predicting the probability of choosing the risky option in a trial.

**Table 2:** Result of the logistic regression with subject level random effects.

Independent variables	Dependent variable	
	Risky choice	
	Model 1.1	Model 1.2
Sweet-liking	−1.68*	−2.53*
	(0.67)	(1.09)
Task order	1.55**	1.58**
	(0.57)	(0.58)
Risky reward	0.02***	0.02***
	(0.001)	(0.001)
Probability of risky reward = 0.5	2.90***	2.78***
	(0.17)	(0.19)
Probability of risky reward = 0.75	4.46***	4.33***
	(0.20)	(0.22)
Sweet-liking ⨯ Risky reward		0.001
		(0.002)
Sweet-liking ⨯ (Probability of risky reward = 0.5)		0.59
		(0.43)
Sweet-liking ⨯ (Probability of risky reward = 0.75)		0.66
		(0.50)
Gender	−0.63	−0.62
	(0.61)	(0.61)
Age	−0.03	−0.03
	(0.07)	(0.07)
Constant	−7.70***	−7.50***
	(1.86)	(1.87)
Observations	2,940	2,940
Akaike Inf. Crit.	2,042.90	2,046.52

Dependent variable-probability to choose risky reward, Experiment 1. Standard errors are shown in brackets. ***p < 0.001, **p < 0.01, *p < 0.05.

Across both models, a higher risky reward (Model 1.1: *β* = 0.02, *p* < 0.001; Model 1.2: *β* = 0.02, *p* < 0.001) and a higher winning probability (Model 1.1: *β* = 4.46, *p* < 0.001; Model 1.2: *β* = 4.33, *p* < 0.001) were associated with a greater probability of choosing the risky option, as expected. Sweet-liking status was also a significant predictor of risky choices, indicating that sweet-likers were less likely to choose the risky option compared to sweet-dislikers (Model 1.1: *β* = −1.68, *p* < 0.01; Model 1.2: *β* = −2.53, *p* < 0.02). To further assess the contribution of sweet-liking status to risky choice, we additionally estimated McFadden’s pseudo-R^2^ for both models. In Model 1.1, the full model explained 45.3% of the variance in risky choices (McFadden’s pseudo-*R*^2^ = 0.453), while sweet-liking status accounted for an additional 0.2% of the variance beyond the other predictors (Δ*R*^2^ = 0.002; *χ*^2^(1) = 5.98, *p* = 0.014). In Model 1.2, the combined contribution of sweet-liking status and its interactions with reward magnitude and winning probability was also 0.2% (Δ*R*^2^ = 0.002; *χ*^2^(4) = 8.35, *p* = 0.079). The Akaike Information Criterion indicated a better fit for Model 1.1 than for Model 1.2.

#### BART

**Figure 4:**
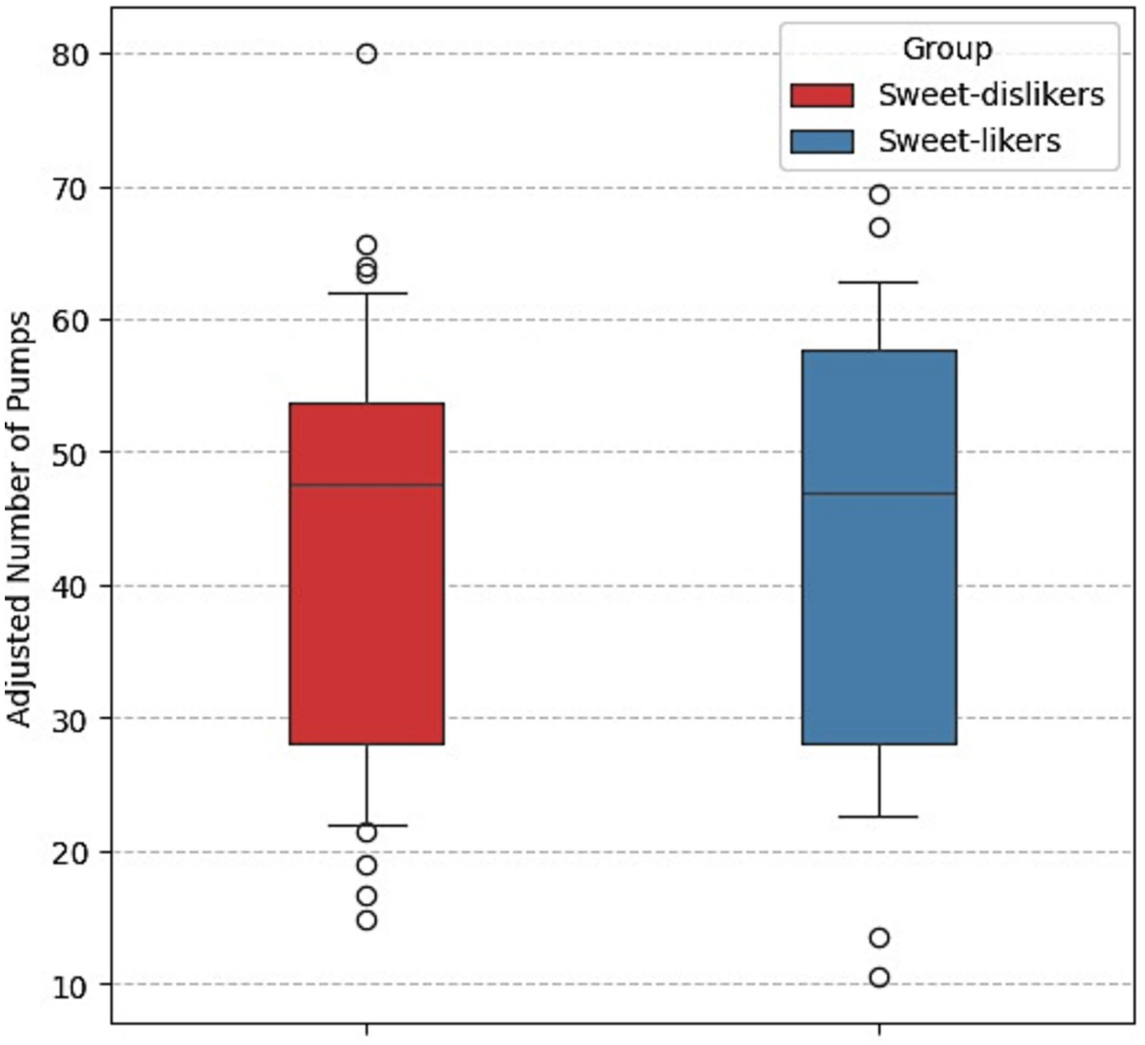
Comparison of mean number of adjusted pumps between sweet-liking groups, Experiment 1.

Figure 4 illustrates the comparison of the mean number of adjusted pumps in the BART between the two sweet-liking groups. Sweet-likers and sweet-dislikers showed a similar average number of adjusted pumps, indicating no clear difference in risk-taking behavior on the BART between them.

Table 3 presents the estimation results for a linear regression predicting the adjusted number of pumps in the BART task. The model showed that none of the predictors were significant (all *p*-values > 0.1), indicating no difference in BART risk taking between the sweet-liking groups.

**Table 3:** Result of the linear regression.

Independent variables	Dependent variable
	Mean number of adjusted pumps
	Model 1.3
Sweet-liking	0.19
	(6.04)
Task order	0.04
	(5.18)
Gender	−2.51
	(5.50)
Age	0.04
	(0.66)
Constant	43.91*
	(16.61)
Observations	49
Adjusted	−0.09

Dependent variable-mean number of adjusted pumps, Experiment 1. Standard errors are shown in brackets. ***p < 0.001, **p < 0.01, *p < 0.05.

### Discussion

Experiment 1 examined whether individual differences in sweet taste preferences are associated with risk-taking behavior and whether this relationship depends on the type of risk-taking task. Specifically, we investigated whether the sweet-liking phenotype predicts performance in two types of decision-making tasks: the lottery choice task and the BART. To the best of our knowledge, this is the first study to examine this relationship.

The experimental results showed that sweet-likers were more risk averse in the lottery choice task. Specifically, sweet-likers were less likely to choose risky options compared to sweet-dislikers. Furthermore, this difference was not driven by differences in reward valuation or sensitivity to probability, as indicated by the insignificant interaction effects of the sweet-liking phenotype with these variables.

However, no significant differences were found between sweet-likers and sweet-dislikers in the BART. This suggests that the relationship between sweet-liking and risk-taking may be influenced by the structure of the decision-making task. In the lottery choice task, participants make a single binary choice between a risky and a safe option with explicitly stated probabilities and reward magnitudes. This structure implies evaluation of reward value under known uncertainty. In contrast, the BART involves sequential decisions under experiential uncertainty, where risk gradually accumulates and is inferred through feedback rather than explicitly stated probabilities. Performance in the BART is therefore more strongly influenced by learning dynamics and emotional reactions to experienced gains and losses (Jepma et al., 2022; Schonberg et al., 2011). The sweet-liking phenotype has been linked to individual differences in reward-related processes (Kampov-Polevoy et al., 2003; Weafer et al., 2014). Given this association, sweet-liking may be more closely related to decision contexts that emphasize explicit reward magnitude and value comparison, as in the lottery task, rather than to paradigms that primarily engage experiential learning and uncertainty tolerance, such as the BART. Additionally, the BART paradigm implies that both the value of the safe option and the risk of burst increase simultaneously with each balloon pump. Since the sweet-liking preferences have been linked to the activity of the opioid system which is related to both the assessment of positive as well as negative outcomes, it is possible that the direct relationship between the sweet-liking phenotype and risk-taking behavior is obscured in the contexts where risks and rewards are correlated.

A recent meta-analysis reported small effect sizes for the BART across several metrics, including known-groups validity (*d* = 0.10), convergent validity with other risk-taking measures (*r* = 0.00), and external validity (*r* = 0.02), which may explain the lack of significant group differences in the BART task (Frey et al., 2025).

Moreover, in the regression analysis for the lottery choice task, we observed a main effect of sweet-liking, but no significant interaction between sweet-liking and reward magnitude or probability. This suggests that sweet-likers may have a general bias toward the safe option that is not driven by differences in reward valuation or sensitivity.

In addition, our results may be partially influenced by the task order, as almost half of the participants (24 out of 49) completed the lottery choice task and the BART after the sweet-taste liking test. To account for this, we included task order as a control variable in the regression analysis. We found that it was statistically significant for the lottery choice task. This suggests that the order of tasks had an effect on participants’ decision-making. One possible explanation is that the sweet-taste liking test may have triggered emotional reactions that affected participants’ later decisions. For instance, it is possible that sweet-likers reacted more positively when the sweet-taste liking test was presented first, while sweet-dislikers found it unpleasant. This may have introduced a systematic bias into their decision-making. Although we added the task order as a control variable in a regression analysis, it may not have fully eliminated the potential influence of task sequence on the results.

Moreover, it is important to note that during the sweet-taste liking test in Experiment 1, participants rinsed their mouths with water before rating each solution. This differs from the standard procedure and may have slightly affected their absolute ratings of sweetness and liking. However, this seemed to have no effect on the overall results because sweet-likers were identified as those who rated the highest concentration of sucrose solution as the most pleasant, which implies that the relative ranking of the solutions would likely remain the same. In Experiment 2, we took this limitation into account by revising the order so that participants rated the solution first and then rinsed their mouths with water.

## Experiment 2

### Materials and methods

#### Participants

The study was conducted following the same ethical guidelines and regulations as outlined in Experiment 1. A new sample of 100 healthy participants (56 females, mean age = 22.92 years, SD = 5.03) was recruited via the university’s database and online advertisements. Eligibility and exclusion criteria were identical to those in Experiment 1. All participants provided written informed consent prior to the beginning of the experiment. All participants were compensated with 250 monetary units in local currency (~8.5 USD by purchasing power parity at the time of the experiment).

#### Experimental procedure

Participants attended a 1.5-h experimental session, following the same general procedures as in Experiment 1. Experiment 2 differed from Experiment 1 in three major ways. First, the BART task was not used, since no significant association was found between BART performance and the sweet-liking phenotype. Second, in Experiment 2, in addition to the lottery choice task participants completed the delay discounting task to measure their monetary reward impulsivity. Third, the order of the tasks was fixed so that the sweet-taste liking test was always completed after the behavioral tasks, since Experiment 1 showed that participants’ choices differed depending on whether they completed the task before or after the sweet-taste liking test. For the delay discounting and the lottery choice task, a block design was used. Across 10 blocks, participants first completed 6 lottery choice trials, followed by 17 delay discounting trials per block.

Following the behavioral tasks and the sweet-taste liking test, participants completed a set of questionnaires including the Sensation Seeking and the Novelty Seeking scales as in Experiment 1. Additionally we added a self assessment of risky behavior on a scale from 0 (“I consider myself not risky at all”) to 10 (“I consider myself very risky”), since prior research has shown that this self-report measure is a valid indicator of actual risk behavior that captures individual differences in risk preferences (Dohmen et al., 2011; Szrek et al., 2012). We also administered the Barratt Impulsiveness Scale (BIS) as a reliable and widely used measure for assessing impulsivity traits (Barratt, 1959).

#### Measures

##### Sweet-taste liking task

The procedure in Experiment 2 was almost the same as in Experiment 1. However, in Experiment 1, participants first rinsed their mouths with water after tasting each sucrose solution and then rated its sweetness and pleasantness. In Experiment 2, participants rated the solution first and then rinsed their mouths with water.

##### Lottery choice task

In Experiment 2, the lottery choice task followed the same design as in Experiment 1 but was mixed with delay discounting trials within a block. Participants were compensated based on their choice in one randomly selected trial, and were informed about this payment scheme in the experimental instructions.

##### Delay discounting task

**Figure 5:**
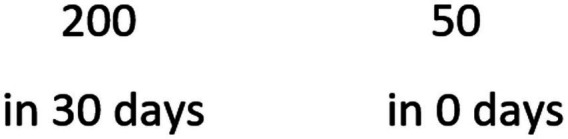
An example of a trial in the delay discounting task. A participant is offered a choice between 200 MU in 30 days and 50 MU immediately.

Reward impulsivity was measured using the delay discounting task. This procedure evaluates an individual’s preference for sooner, smaller rewards over larger, delayed ones. The delay discounting task included 170 trials organized into 10 blocks of 17 trials each. In each trial, participants made choices between a smaller, sooner monetary reward (50, 100, or 150 MU) available after 0, 2, or 30 days and a larger, delayed reward (ranging from 100 to 500 MU in increments of 50) available after 2, 14, 30, 60, or 90 days. Importantly, in our task, participants encountered two types of delay discounting trials. “Immediate” trials implied that the sooner reward was offered immediately, i.e., the time delay for the sooner option was equal to zero. “Non-immediate” trials implied that both options were delayed in time, i.e., the time delay for both rewards was greater than zero. The positions of the sooner and delayed reward options were randomized across trials. Each option could appear on either the left or the right side of the screen. After each block of the delay discounting task, participants completed a block of trials from the lottery choice task. This block structure continued until all trials were completed. The sequence of delays, rewards, and the order of blocks were randomized for each participant. An example delay discounting task trial is shown in Figure 5.

At the end of the experiment, one trial out of the 170 trials was randomly selected, and participants were paid based on the option they chose in this randomly selected trial, in addition to the compensation for participating in the experiment.

#### Data analysis

For between-group mean comparisons on perceived sweetness and liking of the sucrose solutions, as well as the questionnaire scores in the SS, the NS, and the BIS-11 in Experiment 2, we followed the same procedure as in Experiment 1. Since the assumption of normality did not hold for the risk self-assessment, the Wilcoxon rank-sum test was used to assess differences between the SL and SDL groups on these measures.

To evaluate the relationship between the sweet-liking phenotype and risk-taking, the data from the lottery choice task were analyzed using the same logit models with subject-level random effects as in Experiment 1. The only difference was that the variable “task order” was not included in the regression models, as all participants in Experiment 2 completed the decision-making tasks before the sweet-taste liking test. The following two regression models (with and without interaction terms) were estimated (Equations 2.1 and 2.2):


$$\begin{array}{l} \mathrm{Pr}{\left(\text{choose risky option}\right)}_{\mathrm{ij}}=\beta_{0}+\beta_{1}\text{Sweet likin}g_{i}+\beta_{2}\text{Risky rewar}d_{\mathrm{ij}}+\beta_{3}{\left(\text{Probability of risky reward}=0.5\right)}_{\mathrm{ij}}+\\ \;\;\;\;\;\;\;\;\;\;\;\;\;\;\;\;\;\;\;\;\;\;\;\;\;\;\;\;\;\;\;\;\;\;\;\;\;\;\;\;\;\;\;\;\;\;\;\;\;\;\;\;\;\;\;\;\;\;\;\;\;\;\;\beta_{4}{\left(\text{Probability of risky reward}=0.75\right)}_{\mathrm{ij}}+\gamma \cdot \text{Control}s_{i}+\varepsilon_{\mathrm{ij}} \end{array}$$ (2.1)

$$\begin{array}{l} \mathrm{Pr}{\left(\text{choose risky option}\right)}_{\mathrm{ij}}=\beta_{0}+\beta_{1}\text{Sweet likin}g_{i}+\beta_{2}\text{Risky rewar}d_{\mathrm{ij}}+\beta_{3}{\left(\text{Probability of risky reward}=0.5\right)}_{\mathrm{ij}}+\\ \;\;\;\;\;\;\;\;\;\;\;\;\;\;\;\;\;\;\;\;\;\;\;\;\;\;\;\;\;\;\;\;\;\;\;\;\;\;\;\;\;\;\;\;\;\;\;\;\;\;\;\;\;\;\;\;\;\;\;\;\;\;\beta_{4}{\left(\text{Probability of risky reward}=0.75\right)}_{\mathrm{ij}}+\beta_{5}\text{Sweet likin}g_{i}\times \text{Risky rewar}d_{\mathrm{ij}}+\\ \;\;\;\;\;\;\;\;\;\;\;\;\;\;\;\;\;\;\;\;\;\;\;\;\;\;\;\;\;\;\;\;\;\;\;\;\;\;\;\;\;\;\;\;\;\;\;\;\;\;\;\;\;\;\;\;\;\;\;\;\;\beta_{6}\text{Sweet likin}g_{i}\times {\left(\text{Probability of risky reward}=0.5\right)}_{\mathrm{ij}}+\beta_{7}\text{Sweet likin}g_{i}\times \\ \;\;\;\;\;\;\;\;\;\;\;\;\;\;\;\;\;\;\;\;\;\;\;\;\;\;\;\;\;\;\;\;\;\;\;\;\;\;\;\;\;\;\;\;\;\;\;\;\;\;\;\;\;\;\;\;\;\;\;\;\;{\left(\text{Probability of risky reward}=0.75\right)}_{\mathrm{ij}}+\gamma \cdot \text{Control}s_{i}+\varepsilon_{\mathrm{ij}} \end{array}$$ (2.2)

In the delay discounting task, we analyzed the probability of choosing the delayed option in each trial. We first used a regression model without any interaction terms to determine whether sweet-likers had any bias toward the sooner, smaller option regardless of option characteristics.

In the first model, the sweet-liking phenotype was included as the main variable of interest. Additional variables included the size of the sooner, smaller reward and the larger, later reward, the time delay for the delayed option, and a binary variable indicating reward immediacy (equal to 1 when the sooner reward was immediate and 0 otherwise). Age and gender (1 for female, 0 for male) were included as control variables. Subject-level random effects were included in this and all other models to take into account unobserved individual differences. The model took the following form (Equation 2.3):


$$\begin{array}{l} \mathrm{Pr}{\left(\text{choose delayed option}\right)}_{\mathrm{ij}}=\beta_{0}+\beta_{1}\text{Sweet likin}g_{i}+\beta_{2}\text{Sooner rewar}d_{\mathrm{ij}}+\beta_{3}\text{Delayed rewar}d_{\mathrm{ij}}+\beta_{4}\text{Delay in}\;\mathrm{day}s_{\mathrm{ij}}+\\ \;\;\;\;\;\;\;\;\;\;\;\;\;\;\;\;\;\;\;\;\;\;\;\;\;\;\;\;\;\;\;\;\;\;\;\;\;\;\;\;\;\;\;\;\;\;\;\;\;\;\;\;\;\;\;\;\;\;\;\;\;\;\;\;\;\;\;\;\;\;\;\beta_{5}\text{Trial is immediat}e_{\mathrm{ij}}\;(1=\mathrm{yes},0=\mathrm{no})+\gamma \cdot \text{Control}s_{i}+\varepsilon_{\mathrm{ij}} \end{array}$$ (2.3)

To evaluate the specific pathways through which sweet-liking phenotype might affect intertemporal choice, we estimated the model which included the interaction terms of the sweet-liking phenotype with reward amount, reward immediacy and the time delay toward a later option. The model took the following form (Equation 2.4):


$$\begin{array}{l} \mathrm{Pr}{\left(\text{choose delayed option}\right)}_{\mathrm{ij}}=\beta_{0}+\beta_{1}\text{Sweet likin}g_{i}+\beta_{2}\text{Sooner rewar}d_{\mathrm{ij}}+\beta_{3}\text{Delayed rewar}d_{\mathrm{ij}}+\beta_{4}\text{Delay in}\;\mathrm{day}s_{\mathrm{ij}}+\\ \;\;\;\;\;\;\;\;\;\;\;\;\;\;\;\;\;\;\;\;\;\;\;\;\;\;\;\;\;\;\;\;\;\;\;\;\;\;\;\;\;\;\;\;\;\;\;\;\;\;\;\;\;\;\;\;\;\;\;\;\;\;\;\;\;\;\;\;\;\;\beta_{5}\text{Trial is immediat}e_{\mathrm{ij}}\;(1=\mathrm{yes},0=\mathrm{no})+\beta_{6}\text{Sweet likin}g_{i}\times \text{Sooner rewar}d_{\mathrm{ij}}+\\ \;\;\;\;\;\;\;\;\;\;\;\;\;\;\;\;\;\;\;\;\;\;\;\;\;\;\;\;\;\;\;\;\;\;\;\;\;\;\;\;\;\;\;\;\;\;\;\;\;\;\;\;\;\;\;\;\;\;\;\;\;\;\;\;\;\;\;\;\;\;\beta_{7}\text{Sweet likin}g_{i}\times \text{Delayed rewar}d_{\mathrm{ij}}+\beta_{8}\text{Sweet likin}g_{i}\times \text{Delay in}\;\mathrm{day}s_{\mathrm{ij}}+\\ \;\;\;\;\;\;\;\;\;\;\;\;\;\;\;\;\;\;\;\;\;\;\;\;\;\;\;\;\;\;\;\;\;\;\;\;\;\;\;\;\;\;\;\;\;\;\;\;\;\;\;\;\;\;\;\;\;\;\;\;\;\;\;\;\;\;\;\;\;\beta_{9}\text{Sweet likin}g_{i}\times \text{Trial is immediat}e_{\mathrm{ij}}\;(1=\mathrm{yes},0=\mathrm{no})+\gamma \cdot \text{Control}s_{i}+\varepsilon_{\mathrm{ij}} \end{array}$$ (2.4)

To test the hypothesis that the relationship between the sweet-liking preferences and impulsive choices may be influenced by individual risk preferences, we estimated a third model which included the number of risky choices (the “number of risky choices” variable) made in the lottery choice task by each participant as an additional predictor in the regression model. The model was as follows (Equation 2.5):


$$\begin{array}{l} \mathrm{Pr}{\left(\text{choose delayed option}\right)}_{\mathrm{ij}}=\beta_{0}+\beta_{1}\text{Sweet likin}g_{i}+\beta_{2}\text{Sooner rewar}d_{\mathrm{ij}}+\beta_{3}\text{Delayed rewar}d_{\mathrm{ij}}+\\ \;\;\;\;\;\;\;\;\;\;\;\;\;\;\;\;\;\;\;\;\;\;\;\;\;\;\;\;\;\;\;\;\;\;\;\;\;\;\;\;\;\;\;\;\;\;\;\;\;\;\;\;\;\;\;\;\;\;\;\;\;\;\;\;\;\;\;\;\;\;\beta_{4}\text{Delay in}\;\mathrm{day}s_{\mathrm{ij}}+\beta_{5}\text{Trial is immediat}e_{\mathrm{ij}}\;(1=\mathrm{yes},0=\mathrm{no})=0)+\\ \;\;\;\;\;\;\;\;\;\;\;\;\;\;\;\;\;\;\;\;\;\;\;\;\;\;\;\;\;\;\;\;\;\;\;\;\;\;\;\;\;\;\;\;\;\;\;\;\;\;\;\;\;\;\;\;\;\;\;\;\;\;\;\;\;\;\;\;\;\beta_{6}\text{Sweet likin}g_{i}\times \text{Sooner rewar}d_{\mathrm{ij}}+\beta_{7}\text{Sweet likin}g_{i}\times \text{Delayed rewar}d_{\mathrm{ij}}+\\ \;\;\;\;\;\;\;\;\;\;\;\;\;\;\;\;\;\;\;\;\;\;\;\;\;\;\;\;\;\;\;\;\;\;\;\;\;\;\;\;\;\;\;\;\;\;\;\;\;\;\;\;\;\;\;\;\;\;\;\;\;\;\;\;\;\;\;\;\;\beta_{8}\text{Sweet likin}g_{i}\times \text{Delay in}\;\mathrm{day}s_{\mathrm{ij}}+\beta_{9}\text{Sweet likin}g_{i}\times \text{Trial is immediat}e_{\mathrm{ij}}\;(1=\mathrm{yes},0=\mathrm{no})+\\ \;\;\;\;\;\;\;\;\;\;\;\;\;\;\;\;\;\;\;\;\;\;\;\;\;\;\;\;\;\;\;\;\;\;\;\;\;\;\;\;\;\;\;\;\;\;\;\;\;\;\;\;\;\;\;\;\;\;\;\;\;\;\;\;\;\;\;\;\;\beta_{10}\text{Number of risky choice}s_{i}+\beta_{11}\text{Trial is immediat}e_{\mathrm{ij}}\;(1=\mathrm{yes},0=\mathrm{no})\times \\ \;\;\;\;\;\;\;\;\;\;\;\;\;\;\;\;\;\;\;\;\;\;\;\;\;\;\;\;\;\;\;\;\;\;\;\;\;\;\;\;\;\;\;\;\;\;\;\;\;\;\;\;\;\;\;\;\;\;\;\;\;\;\;\;\;\;\;\;\;\text{Number of risky choice}s_{i}+\gamma \cdot \text{Control}s_{i}+\varepsilon_{\mathrm{ij}} \end{array}$$ (2.5)

Importantly, we did not use the traditional approaches to evaluating delay discounting, which consist in computing the delay discounting coefficient using the hyperbolic function (McKerchar et al., 2009; Mitchell et al., 2015) or a model-free measure such as the area under the discounting curve (AUC) (Mitchell et al., 2015; Myerson et al., 2001; Weafer et al., 2014). Although these approaches are frequently used to characterize the degree of delay discounting at the individual level, they do not allow to evaluate the contribution of specific option characteristics to the tendency toward impulsive choice. In particular, they do not allow us to directly evaluate whether sweet-liking increases sensitivity to various choice components, such as reward amount and reward immediacy, which is central to our research question. The regression analysis has two major advantages in this respect. First, this approach does not depend on the assumptions about a specific structural model of delay discounting (such as hyperbolic, quasi-hyperbolic, etc.), and second, it allows for the direct evaluation of the sweet-liking phenotype relation to intertemporal choices on a trial-by-trial level, as well as its interaction with separate option characteristics such as reward amount, reward immediacy and delay length.

### Results

#### Sweet-taste liking test

Participants were classified as sweet-likers or sweet-dislikers based on their liking ratings for the highest sucrose concentration (0.83 M). Twenty-eight participants were classified as sweet-likers (16 males and 12 females); the remaining seventy-two (28 males and 44 females) were classified as sweet-dislikers. Although this proportion of 28 percent sweet-likers is somewhat lower than the 37 percent previously reported by Weafer et al. (2014), it reflects the same general finding that sweet-likers are less prevalent in the population than sweet-dislikers.

Table 4 summarizes the group means for perceived sweetness and liking across the five sucrose concentrations (0.05, 0.10, 0.21, 0.42, and 0.83 M). Sweetness ratings did not significantly differ between the groups at any concentration, indicating that group differences in liking were not driven by differences in perceived sweetness (all *p*-values > 0.34).

**Table 4:** Mean sweetness and liking ratings across concentrations between sweet-liking groups, Experiment 2.

Measure	Phenotype	Concentration				
		0.05	0.10	0.21	0.42	0.83
Mean sweetness	Sweet-dislikers	23.03	40.47	79.36	125.08	161.75
	Sweet-likers	28.79	35.86	74.57	124.43	159.07
	*p*-value	0.60	0.34	0.42	0.96	0.36
Mean liking	Sweet-dislikers	8.28	9.36	15.28	6.69	−16.42
	Sweet-likers	−7.64	−5.93	12	31.07	51.07
	*p*-value	0.01	0.003	0.34	0.001	0.00

**Figure 6:**
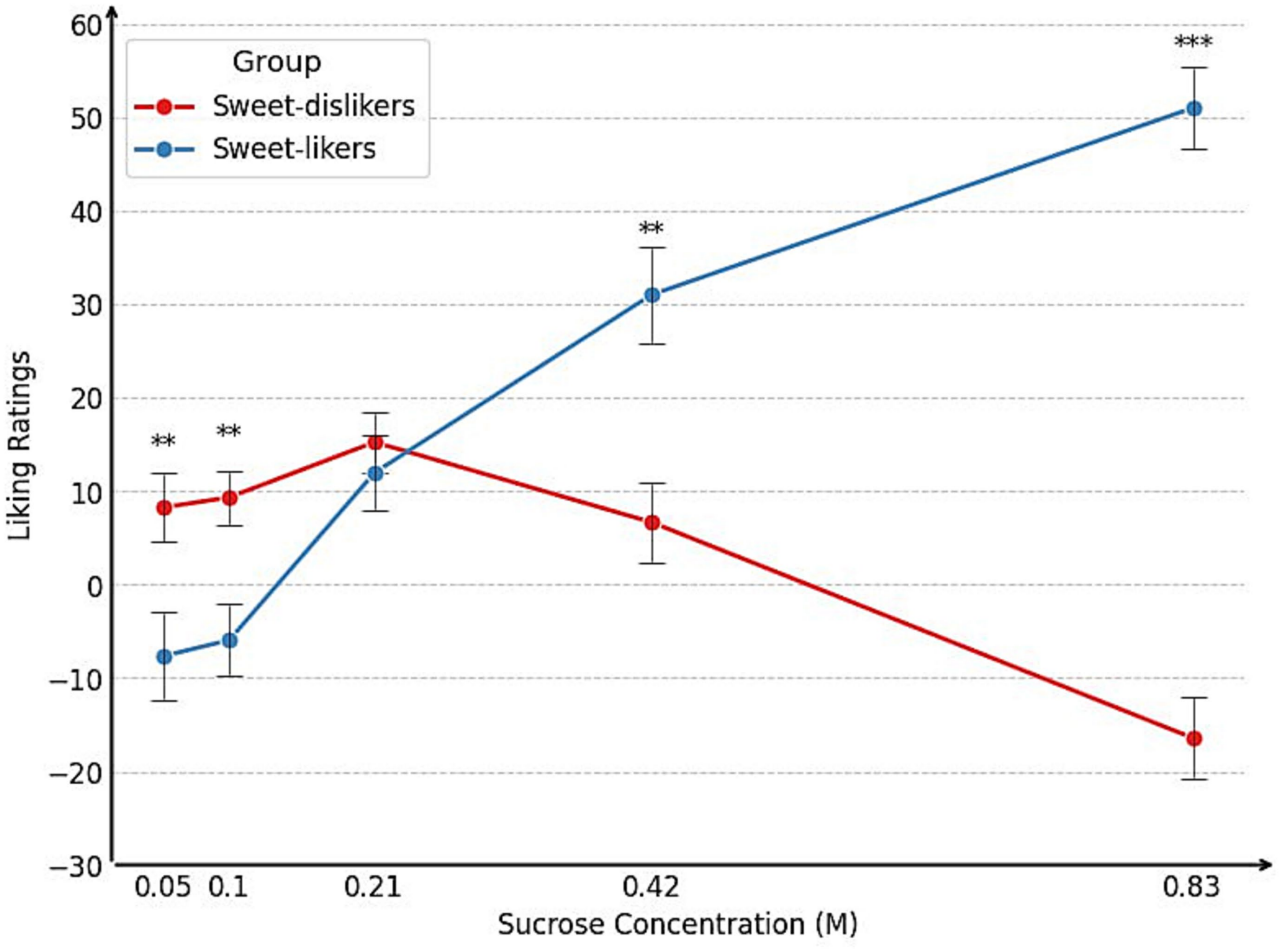
Mean liking across concentrations between sweet-liking groups, Experiment 2. ***p < 0.001, **p < 0.01, *p < 0.05.

However, sweet-likers gave significantly lower liking ratings for the low sucrose concentrations but significantly higher ratings for the high concentrations compared to sweet-dislikers. This pattern is clearly illustrated in Figure 6 and is consistent with the findings from Experiment 1. For sweet-likers, liking ratings increased monotonically with sucrose concentration. In contrast, sweet-dislikers showed a non-monotonic pattern: liking rose and peaked at 0.21 M, then declined sharply at higher concentrations. More detailed information on the distribution of responses to the sweet-liking task across sweet-liking groups can be found in Supplementary material.

#### Personality scales

Scores on the Sensation Seeking, Novelty Seeking, Risk self assessment and BIS-11 scales did not significantly differ between sweet-likers and sweet-dislikers (*p* = 0.06; *p* = 0.71, *p* = 0.72; *p* = 0.96 respectively). Detailed analyses of these measures are reported in Supplementary material.

#### Lottery choice task

**Figure 7:**
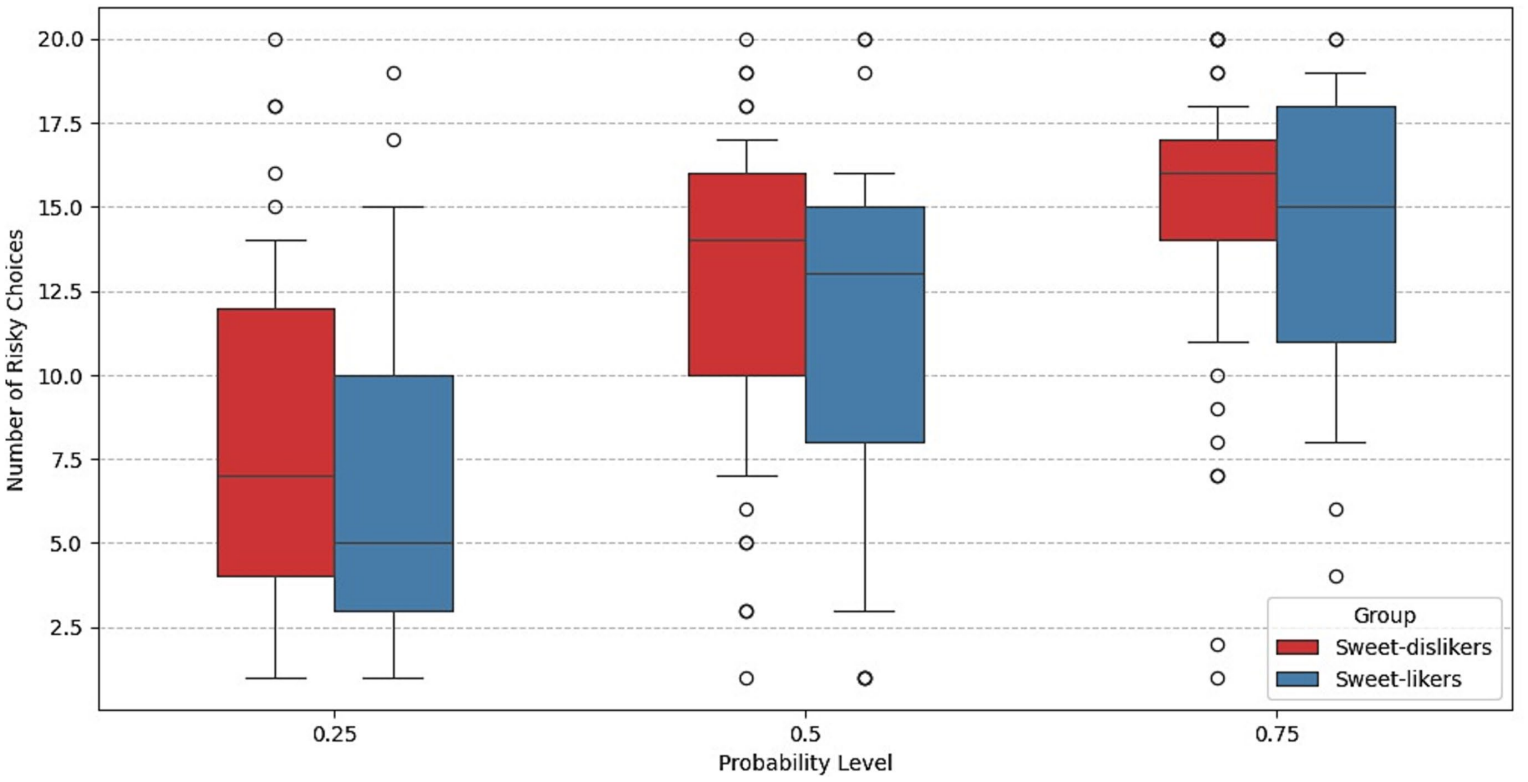
Number of risky choices in the lottery choice task across trials with different probabilities in Experiment 2.

Figure 7 presents the average counts of risky choices by group across the three winning probabilities. For both groups, the number of risky choices increases with the probability of obtaining the risky payoff.

Table 5 presents the results from the logistic regression model with subject-level random effects predicting the probability of choosing the risky option in a trial.

**Table 5:** Results of the logistic regression with subject-level random effects.

Independent variables	Dependent variable	
	Risky choice	
	Model 2.1	Model 2.2
Sweet-liking	−0.60	0.16
	(0.45)	(0.59)
Risky reward	0.01***	0.01***
	(0.00)	(0.001)
Probability of risky reward = 0.5	2.57***	2.78***
	(0.10)	(0.13)
Probability of risky reward = 0.75	3.58***	3.69***
	(0.12)	(0.14)
Sweet-liking ⨯ Risky reward		−0.001
		(0.001)
Sweet-liking ⨯ (Probability of risky reward = 0.5)		−0.75***
		(0.23)
Sweet-liking ⨯ (Probability of risky reward = 0.75)		−0.39
		(0.25)
Gender	−0.98*	−0.96*
	(0.42)	(0.42)
Age	−0.02	−0.02
	(0.04)	(0.04)
Constant	−5.25***	−5.41***
	(1.10)	(1.11)
Observations	6,000	6,000
Akaike Inf. Crit.	4,735.09	4,729.57

Dependent variable-probability of choosing the risky reward, Experiment 2. Standard errors are shown in brackets. ***p < 0.001, **p < 0.01, *p < 0.05.

Across both models, higher risky payoff and higher winning probability were associated with a greater probability of choosing the risky option, as expected. Female participants were less likely than males to choose the risky option. Sweet-liking status alone was not a significant predictor, however, at a winning probability equal to 0.50, sweet-likers were less likely to choose the risky option compared to sweet-dislikers (Model 2.2: *β* = −0.75, *p* < 0.001). These findings are consistent with those from Experiment 1, suggesting that sweet-likers demonstrated a preference for less risky options. Importantly, in Experiment 1 sweet-liking status alone was associated with a lower probability of choosing the risky option, whereas in Experiment 2 this effect was observed specifically in trials with 50% winning probability. One possible explanation is the difference in experimental procedure: in Experiment 1, task order varied across participants and was itself a significant predictor of risky choice, whereas in Experiment 2 task order was fixed. In addition, the smaller sample size in Experiment 1 may have limited statistical power to detect the interaction between sweet-liking and winning probability. More detailed information on the distribution of responses to the lottery choice task across sweet-liking groups can be found in Supplementary material.

#### Delay discounting task

Table 6 presents the results from the logistic regression model with subject-level random effects predicting the probability of choosing the delayed option.

**Table 6:** Results of the logistic regression with subject-level random effects.

Independent variables	Dependent variable		
	Delayed choice		
	Model 2.3	Model 2.4	Model 2.5
Sweet-liking	1.02	1.75**	1.73*
	(0.61)	(0.67)	(0.68)
Sooner reward	−0.02***	−0.02***	−0.02***
	(0.001)	(0.001)	(0.001)
Delayed reward	0.01***	0.01***	0.01***
	(0.00)	(0.00)	(0.00)
Delay in days	−0.04***	−0.04***	−0.04***
	(0.001)	(0.001)	(0.001)
Trial is immediate (1 = yes, 0 = no)	−1.002***	−0.88***	−1.71***
	(0.07)	(0.07)	(0.20)
Sweet-liking ⨯ Sooner reward		−0.001	−0.001
		(0.002)	(0.002)
Sweet-liking ⨯ Delayed reward		−0.00	−0.001
		(0.001)	(0.001)
Sweet-liking ⨯ Delay in days		−0.003	−0.003
		(0.003)	(0.003)
Sweet-liking ⨯ Trial is immediate (1 = yes, 0 = no)		−0.53***	−0.31
		(0.16)	(0.17)
Number of risky choices			0.01
			(0.02)
Trial is immediate (1 = yes, 0 = no) ⨯ Number of risky choices			0.03***
			(0.01)
Gender	−0.42	−0.43	−0.24
	(0.55)	(0.55)	(0.57)
Age	−0.08	−0.08	−0.08
	(0.06)	(0.06)	(0.05)
Constant	5.14***	4.98***	4.52**
	(1.44)	(1.44)	(1.65)
Observations	17,000	17,000	17,000
Akaike Inf. Crit.	9,104.55	9,099.77	9,083.12

Dependent variable-the probability of choosing the delayed reward, Experiment 2. Standard errors are shown in brackets. ***p < 0.001, **p < 0.01, *p < 0.05.

The results showed that longer delays (Model 2.3: *β* = −0.04, *p* < 0.001; Model 2.4: *β* = −0.04, *p* < 0.001) and larger immediate amounts (Model 2.3: *β* = −0.021, *p* < 0.001; Model 2.4: *β* = −0.02, *p* < 0.001) reduced the probability of choosing the delayed option, whereas larger delayed amounts increased it (Model 2.3: *β* = 0.01, *p* < 0.001; Model 2.4: *β* = 0.01, *p* < 0.001). In the immediate trials, the probability of choosing the delayed option was reduced compared to the non-immediate trials (Model 2.3: *β* = −1.002, *p* < 0.001).

When the immediate option was available, sweet-likers were significantly more likely to choose the immediate option compared to sweet-dislikers (Model 2.4: *β* = −0.53, *p* < 0.001). However, in the non-immediate trials, this result was reversed: sweet-likers were significantly more likely to choose the delayed option, as indicated by the significant positive coefficient for the sweet-liking variable (Model 2.4: *β* = 1.75, *p* = 0.009). This observation is consistent with the pattern of preference reversals, which are a characteristic feature of impulsive behavior (Sharma and Khan, 2021).

However, no other interaction terms between the sweet-liking phenotype and the size of the reward or the length of the delay were significant, suggesting that the increased impulsivity of sweet-likers was primarily driven by a bias toward the immediate option and was unrelated to other option characteristics. We further tested whether this bias could be explained by the risks associated with the delay, given that sweet-likers demonstrated greater risk avoidance compared to sweet-dislikers. If this is indeed the case, then we would expect that, when their risk preferences from the lottery choice task are taken into account, the association between the sweet-liking phenotype and impulsivity would be reduced or would become non-significant. To test this hypothesis, we estimated Model 2.5, which included an additional explanatory variable, Number of risky choices, representing the number of risky choices that a participant made in the lottery choice task. This variable was included both independently and in an interaction term with the binary variable indicating immediate trials.

We observed that the number of risky choices was a significant predictor of choosing the delayed option, particularly in the presence of immediate options. Moreover, we observed that although in Model 2.4 the sweet-liking phenotype was highly significantly associated with impulsive choices in the immediate trials, adding the overall risk propensity rendered it only marginally significant (Model 2.4: interaction *β* = −0.53, *p* < 0.001; Model 2.5: *β* = −0.31, *p* = 0.06). This result suggests that the overall risk propensity at least partially explains the higher impulsivity observed in sweet-likers compared to sweet-dislikers. More detailed information on the distribution of responses to the delay discounting task across sweet-liking groups can be found in Supplementary material.

### Discussion

Experiment 2 examined whether individual differences in sweet taste preferences are associated with impulsive behavior and whether this relationship can be partially explained by differences in sensitivity to reward size, reward immediacy, or risk preferences between sweet-likers and sweet-dislikers. Supporting the results of Experiment 1, Experiment 2 also showed that sweet-likers are significantly less likely to choose the risky option compared to sweet-dislikers. Overall, these findings are consistent with those from Experiment 1 in terms of the general direction of the effect. However, in Experiment 1, sweet-likers generally preferred the safe option regardless of the reward amount or probability level, while in Experiment 2 the preference for the safe option was mainly observed in trials where the probability of winning was 50 percent. This result may stem from the fact that a 50 percent probability represents the highest level of uncertainty in a lottery. When the probability of winning is relatively low (25 percent), most participants would prefer the safe option, and when the winning probability is relatively high (75 percent), most participants would prefer to play the lottery regardless of their sweet-liking phenotype. Therefore, the differences in risk preferences between the groups would be most significant when the uncertainty is at its highest.

The difference from Experiment 1 may have resulted from differences in the experimental procedures between the two studies. Importantly, unlike in Experiment 2, in the first study, half of the participants completed the lottery choice task before the sweet-liking test, while the other half completed it after the sweet-taste liking test. Further data analysis confirmed that the order of the tasks had a significant effect on participants’ choices, which may have biased their answers in Experiment 1. This was not an issue in Experiment 2, as the order of the tasks was fixed for all participants.

In the delay discounting task, we observed two distinct effects of the sweet-liking phenotype, depending on the type of trial. In trials where both options were delayed, sweet-likers were more likely to choose the option with the longer delay, showing greater willingness to wait. However, in trials where one option was available immediately and the other was delayed, sweet-likers were more likely to choose the immediate reward, showing greater impulsivity compared to sweet-dislikers. This choice pattern is consistent with the phenomenon of preference reversals, whereby an individual demonstrates patience when both rewards are delayed in time, while showing impulsivity when one of the options is available immediately. Preference reversals have been shown to be present in both human and animal models (Hurtado-Parrado et al., 2024), and are associated with preferential visual processing of immediate rewards (Sharma and Khan, 2021). The latter suggests that the sweet-liking phenotype may be particularly associated with an immediacy bias rather than distortions in the valuation of delayed rewards.

Importantly, we did not find any significant interactions between the sweet-liking phenotype and the size of either the immediate or the delayed reward. Therefore, the hypothesis that reward sensitivity is associated with greater impulsivity via an increased preference for larger immediate rewards was not supported. However, this observation may also result from the low variability, particularly in the size of the immediate reward, which took only three different values in our experimental paradigm.

Additionally, we found that when risk preferences are taken into account, the difference between the sweet-liking groups in terms of impulsivity becomes only marginally significant, which suggests that higher impulsivity in sweet-likers might be explained by their lower risk tolerance. Delay discounting tasks inherently involve an element of uncertainty. As shown by Andersen et al. (2008), individuals who are more risk averse tend to show much lower discount rates compared to estimates from earlier studies that assumed risk neutrality. In our study, this could be explained by the fact that for risk-averse individuals, delayed outcomes may feel more uncertain and therefore riskier. In this context, sweet-likers may appear more impulsive simply because they are more risk-averse and tend to avoid uncertainty.

## General discussion

Previous studies have shown that reward sensitivity might be closely linked to impulsivity, but the exact mechanisms of this association remain poorly understood. The aim of the present study was to investigate the pathways through which the preference for sweet taste as a biological representation of reward sensitivity might be linked to impulsive choice, indicated by the preference for a sooner, smaller reward rather than a larger, later reward. We hypothesized that this association might be driven by differences in sensitivity to reward size, reward immediacy, or to risk preferences associated with increased reward sensitivity. We found that across two experiments participants demonstrated that the sweet-liking phenotype was associated with a lower propensity to take risks in the lottery choice task. In the delay discounting task, we showed that the sweet-liking phenotype was associated with an increased probability of choosing the sooner, smaller option, particularly when it was available immediately. However, we did not find any significant interactions between the sweet-liking phenotype and either reward amount or the length of delay. Finally, we observed that taking into account differences in risk preferences rendered the link between sweet-liking and impulsive choice only marginally significant, suggesting that sweet-likers’ greater tendency to choose immediate options is at least partially explained by their risk aversion.

A possible explanation for the link between the sweet-liking phenotype and risk preferences might relate to the biological systems involved. Preferences for sweet taste have been linked to activation of the opioid systems. For instance, Meier et al. (2021) indicated that endogenous opioids can enhance both the pleasure (liking) and motivation (wanting) associated with many types of rewards, including sweet taste. While the opioid system is traditionally considered in terms of reward sensitivity and the experience of pleasure (liking), recent research suggests that it also plays a role in the avoidance of negative or uncertain outcomes. For example, receptor binding studies have found that the amygdala and insular cortex have one of the highest concentrations of mu-opioid receptors (MORs) in the brain (Leppä et al., 2006). Both amygdala and insular cortex are associated with the evaluation of threats and uncertainty. Sarinopoulos et al. (2010) have shown that bilateral mid-insula and amygdala responses to aversive stimuli after uncertain cues. Moreover, in the amygdala, bilateral activation has been observed in response to cues related to threat avoidance and escape cues (Schlund and Cataldo, 2010). Hence, it is possible that sweet-liking preferences, reflecting heightened opioid sensitivity, enhance not only the sensitivity to rewarding stimuli but also the aversiveness of uncertain or threatening outcomes. In the present study, we used lotteries only with non-negative outcomes. However, it is still possible that a sure option was considered as a reference point relative to which a zero outcome in a risky option would be considered as a loss (Kahneman and Tversky, 1979). If the sweet-liking preferences are related to the aversion toward negative outcomes, this might explain the relationship observed in our study.

It is important to note that some studies where reward sensitivity was measured using questionnaires report mixed evidence on the links with the risk-taking behavior. Some studies indicate that more reward sensitive individuals are more likely to engage in risky behaviors, such as substance use (Simons and Arens, 2007), reckless driving (Greening and Stoppelbein, 2000; Scott-Parker et al., 2013), or gambling (Gaher et al., 2015). However, other studies where reward sensitivity was represented by the BAS Reward Responsiveness subscale of the BIS/BAS questionnaire showed the opposite pattern whereby higher reward sensitivity was negatively correlated with engaging in risky health behaviors (Voigt et al., 2009; Wu et al., 2021), and may even be associated with positive effects on psychological wellbeing (Taubitz et al., 2015), while BAS Fun Seeking was positively associated with risk-taking tendencies (Shao et al., 2025). Importantly, in our study, we did not find any significant differences between the sweet-likers and sweet-dislikers in terms of the Novelty seeking, Barratt impulsiveness scale, Sensation seeking, or risk self-assessment (see Supplementary material). Our results highlight that the link between reward sensitivity and risk taking may greatly depend on the specific measure of reward sensitivity that is being used, and the divergence may exist between the physiological and psychological measures of reward sensitivity.

Mixed findings can also be seen in the literature examining the relationship between reward sensitivity and delay discounting. For example, Pornpattananangkul and Nusslock (2016) reported that elevated reward-related EEG activity was associated with a greater preference for larger but delayed rewards, whereas in our study sweet-liking participants were more likely to prefer the smaller but immediate option. One possible explanation is that these studies relied on different measures that may capture different aspects of reward-related processing. Pornpattananangkul and Nusslock (2016) used EEG-based indices, whereas the present study relied on the sweet-liking phenotype, a gustatory hedonic marker. Importantly, such inconsistencies may reflect not only differences in measurement, but also the fact that reward sensitivity itself is not a unitary construct. There are also differences in the neural circuitry underlying different types of reward, suggesting that reward sensitivity may be better understood as a domain-specific and multifaceted construct (Stephens et al., 2010).

Interestingly, we observed that it is the preference for reward immediacy that is associated with the sweet-liking phenotype, but not the delay length or the reward amounts. This observation might suggest that reward sensitivity plays a specific role in directing attentional processes toward more salient and more tangible stimuli instead of manifesting as a global increase in the subjective value of larger rewards or as a generalized intolerance to waiting. In other words, it may signify a hyper-responsiveness to the presence of an immediately obtainable reward. Such selectivity aligns with the dual-process and visceral-influence theories of impulsive choice, which posit that tangible rewards engage ‘hot’ affective systems to a greater extent than more distant ones (Nordgren et al., 2007; Steinmetz et al., 2018). Further studies potentially employing eye-tracking techniques are needed to test the attentional direction hypothesis in the context of the sweet-liking preferences and intertemporal choice.

## Data Availability

The datasets presented in this study can be found in online repositories. The names of the repository/repositories and accession number(s) can be found in the article/Supplementary material.
