# Supplementary material for: Sweet-taste liking is associated with preference for less risky and immediate rewards in economic decision-making
Source: Front Psychol. 2026 May 20;17:1764306. doi: 10.3389/fpsyg.2026.1764306 (PMC13231436; doi:10.3389/fpsyg.2026.1764306)
Supplement: Supplementary file 1 [file Supplementary_file_1.DOCX]

Supplementary Material

# Supplementary Tables

|  | **Sensation seeking** | | **Novelty seeking** | |
| --- | --- | --- | --- | --- |
|  | Mean | SD | Mean | SD |
| Sweet-dislikers | 8.25 | 2.14 | 8.83 | 3.08 |
| Sweet-likers | 7.75 | 3.44 | 10 | 3.57 |
| p-value | 0.64 | | 0.33 | |

Table S1. Mean scores on the Sensation Seeking and Novelty Seeking scales between sweet-likers and sweet-dislikers, Experiment 1. P-value corresponds to the Welch’s t-test.

|  | **Sensation seeking** | | | **Novelty seeking** | | | **Risk self assessment** | | | **BIS-11** | | |
| --- | --- | --- | --- | --- | --- | --- | --- | --- | --- | --- | --- | --- |
|  | Mean | SD | Mean | | SD | Mean | | SD | Mean | | SD |  |
| Sweet-dislikers | 7.72 | 2.71 | 9.44 | | 3.5 | 5.94 | | 1.69 | 66.28 | | 9.69 |  |
| Sweet-likers | 8.79 | 2.43 | 9.71 | | 3.18 | 5.75 | | 1.86 | 66.18 | | 9.98 |  |
| p-value | 0.06 | | | 0.71 | | | 0.72 | | | 0.96 | | |

Table S2. Mean scores on the Sensation Seeking, the Novelty Seeking, the Risk self assessment and the BIS-11 scales between sweet-likers and sweet-dislikers, Experiment 2. P-value for the Novelty Seeking, the Sensation seeking and the BIS-11 corresponds to the Welch’s t-test. P-value for the Risk self assessment corresponds to the Wilcoxon rank-sum test.

| **Sure reward** | **Zero reward** | **Probability of zero reward** | **Risky reward** | **Probability of risky reward** | **Percent of risky choices across sweet-dislikers** | **Percent of risky choices across sweet-likers** |
| --- | --- | --- | --- | --- | --- | --- |
| 100 | 0 | 0.25 | 120 | 0.75 | 8.1 | 16.7 |
| 100 | 0 | 0.25 | 140 | 0.75 | 16.2 | 8.3 |
| 100 | 0 | 0.25 | 160 | 0.75 | 24.3 | 8.3 |
| 100 | 0 | 0.25 | 180 | 0.75 | 32.4 | 16.7 |
| 100 | 0 | 0.25 | 200 | 0.75 | 59.5 | 50.0 |
| 100 | 0 | 0.25 | 220 | 0.75 | 62.2 | 50.0 |
| 100 | 0 | 0.25 | 240 | 0.75 | 67.6 | 41.7 |
| 100 | 0 | 0.25 | 260 | 0.75 | 78.4 | 41.7 |
| 100 | 0 | 0.25 | 280 | 0.75 | 81.1 | 58.3 |
| 100 | 0 | 0.25 | 300 | 0.75 | 89.2 | 66.7 |
| 100 | 0 | 0.25 | 320 | 0.75 | 89.2 | 75.0 |
| 100 | 0 | 0.25 | 340 | 0.75 | 94.6 | 75.0 |
| 100 | 0 | 0.25 | 360 | 0.75 | 83.8 | 75.0 |
| 100 | 0 | 0.25 | 380 | 0.75 | 89.2 | 75.0 |
| 100 | 0 | 0.25 | 400 | 0.75 | 97.3 | 91.7 |
| 100 | 0 | 0.25 | 420 | 0.75 | 97.3 | 83.3 |
| 100 | 0 | 0.25 | 440 | 0.75 | 97.3 | 83.3 |
| 100 | 0 | 0.25 | 460 | 0.75 | 97.3 | 83.3 |
| 100 | 0 | 0.25 | 480 | 0.75 | 94.6 | 83.3 |
| 100 | 0 | 0.25 | 500 | 0.75 | 97.3 | 83.3 |
| 100 | 0 | 0.5 | 120 | 0.5 | 8.1 | 0.0 |
| 100 | 0 | 0.5 | 140 | 0.5 | 5.4 | 0.0 |
| 100 | 0 | 0.5 | 160 | 0.5 | 8.1 | 0.0 |
| 100 | 0 | 0.5 | 180 | 0.5 | 13.5 | 8.3 |
| 100 | 0 | 0.5 | 200 | 0.5 | 29.7 | 8.3 |
| 100 | 0 | 0.5 | 220 | 0.5 | 37.8 | 25.0 |
| 100 | 0 | 0.5 | 240 | 0.5 | 54.1 | 25.0 |
| 100 | 0 | 0.5 | 260 | 0.5 | 40.5 | 16.7 |
| 100 | 0 | 0.5 | 280 | 0.5 | 56.8 | 41.7 |
| 100 | 0 | 0.5 | 300 | 0.5 | 67.6 | 50.0 |
| 100 | 0 | 0.5 | 320 | 0.5 | 64.9 | 58.3 |
| 100 | 0 | 0.5 | 340 | 0.5 | 73.0 | 50.0 |
| 100 | 0 | 0.5 | 360 | 0.5 | 73.0 | 58.3 |
| 100 | 0 | 0.5 | 380 | 0.5 | 78.4 | 50.0 |
| 100 | 0 | 0.5 | 400 | 0.5 | 86.5 | 75.0 |
| 100 | 0 | 0.5 | 420 | 0.5 | 83.8 | 66.7 |
| 100 | 0 | 0.5 | 440 | 0.5 | 81.1 | 66.7 |
| 100 | 0 | 0.5 | 460 | 0.5 | 83.8 | 75.0 |
| 100 | 0 | 0.5 | 480 | 0.5 | 83.8 | 66.7 |
| 100 | 0 | 0.5 | 500 | 0.5 | 89.2 | 83.3 |
| 100 | 0 | 0.75 | 120 | 0.25 | 5.4 | 0.0 |
| 100 | 0 | 0.75 | 140 | 0.25 | 2.7 | 0.0 |
| 100 | 0 | 0.75 | 160 | 0.25 | 5.4 | 0.0 |
| 100 | 0 | 0.75 | 180 | 0.25 | 5.4 | 0.0 |
| 100 | 0 | 0.75 | 200 | 0.25 | 8.1 | 0.0 |
| 100 | 0 | 0.75 | 220 | 0.25 | 8.1 | 0.0 |
| 100 | 0 | 0.75 | 240 | 0.25 | 10.8 | 0.0 |
| 100 | 0 | 0.75 | 260 | 0.25 | 16.2 | 0.0 |
| 100 | 0 | 0.75 | 280 | 0.25 | 18.9 | 8.3 |
| 100 | 0 | 0.75 | 300 | 0.25 | 21.6 | 16.7 |
| 100 | 0 | 0.75 | 320 | 0.25 | 29.7 | 0.0 |
| 100 | 0 | 0.75 | 340 | 0.25 | 27.0 | 16.7 |
| 100 | 0 | 0.75 | 360 | 0.25 | 37.8 | 8.3 |
| 100 | 0 | 0.75 | 380 | 0.25 | 35.1 | 0.0 |
| 100 | 0 | 0.75 | 400 | 0.25 | 40.5 | 16.7 |
| 100 | 0 | 0.75 | 420 | 0.25 | 32.4 | 41.7 |
| 100 | 0 | 0.75 | 440 | 0.25 | 37.8 | 25.0 |
| 100 | 0 | 0.75 | 460 | 0.25 | 40.5 | 33.3 |
| 100 | 0 | 0.75 | 480 | 0.25 | 56.8 | 16.7 |
| 100 | 0 | 0.75 | 500 | 0.25 | 59.5 | 50.0 |

Table S3. Distribution of risky choices across all lottery options in the sweet-liking and sweet-disliking groups in the lottery choice task, Experiment 1.

| **Sure reward** | **Zero reward** | **Probability of zero reward** | **Risky reward** | **Probability of risky reward** | **Percent of risky choices across sweet-dislikers** | **Percent of risky choices across sweet-likers** |
| --- | --- | --- | --- | --- | --- | --- |
| 100 | 0 | 0.25 | 120 | 0.75 | 16.7 | 17.9 |
| 100 | 0 | 0.25 | 140 | 0.75 | 16.7 | 28.6 |
| 100 | 0 | 0.25 | 160 | 0.75 | 27.8 | 32.1 |
| 100 | 0 | 0.25 | 180 | 0.75 | 51.4 | 39.3 |
| 100 | 0 | 0.25 | 200 | 0.75 | 62.5 | 50.0 |
| 100 | 0 | 0.25 | 220 | 0.75 | 68.1 | 60.7 |
| 100 | 0 | 0.25 | 240 | 0.75 | 75.0 | 57.1 |
| 100 | 0 | 0.25 | 260 | 0.75 | 70.8 | 57.1 |
| 100 | 0 | 0.25 | 280 | 0.75 | 76.4 | 50.0 |
| 100 | 0 | 0.25 | 300 | 0.75 | 81.9 | 75.0 |
| 100 | 0 | 0.25 | 320 | 0.75 | 83.3 | 82.1 |
| 100 | 0 | 0.25 | 340 | 0.75 | 86.1 | 71.4 |
| 100 | 0 | 0.25 | 360 | 0.75 | 81.9 | 82.1 |
| 100 | 0 | 0.25 | 380 | 0.75 | 84.7 | 85.7 |
| 100 | 0 | 0.25 | 400 | 0.75 | 91.7 | 82.1 |
| 100 | 0 | 0.25 | 420 | 0.75 | 88.9 | 92.9 |
| 100 | 0 | 0.25 | 440 | 0.75 | 90.3 | 89.3 |
| 100 | 0 | 0.25 | 460 | 0.75 | 88.9 | 92.9 |
| 100 | 0 | 0.25 | 480 | 0.75 | 90.3 | 85.7 |
| 100 | 0 | 0.25 | 500 | 0.75 | 93.1 | 96.4 |
| 100 | 0 | 0.5 | 120 | 0.5 | 8.3 | 7.1 |
| 100 | 0 | 0.5 | 140 | 0.5 | 13.9 | 14.3 |
| 100 | 0 | 0.5 | 160 | 0.5 | 18.1 | 14.3 |
| 100 | 0 | 0.5 | 180 | 0.5 | 18.1 | 14.3 |
| 100 | 0 | 0.5 | 200 | 0.5 | 31.9 | 28.6 |
| 100 | 0 | 0.5 | 220 | 0.5 | 45.8 | 42.9 |
| 100 | 0 | 0.5 | 240 | 0.5 | 56.9 | 28.6 |
| 100 | 0 | 0.5 | 260 | 0.5 | 62.5 | 42.9 |
| 100 | 0 | 0.5 | 280 | 0.5 | 61.1 | 46.4 |
| 100 | 0 | 0.5 | 300 | 0.5 | 76.4 | 67.9 |
| 100 | 0 | 0.5 | 320 | 0.5 | 73.6 | 57.1 |
| 100 | 0 | 0.5 | 340 | 0.5 | 75.0 | 71.4 |
| 100 | 0 | 0.5 | 360 | 0.5 | 73.6 | 67.9 |
| 100 | 0 | 0.5 | 380 | 0.5 | 80.6 | 60.7 |
| 100 | 0 | 0.5 | 400 | 0.5 | 86.1 | 71.4 |
| 100 | 0 | 0.5 | 420 | 0.5 | 86.1 | 71.4 |
| 100 | 0 | 0.5 | 440 | 0.5 | 80.6 | 75.0 |
| 100 | 0 | 0.5 | 460 | 0.5 | 88.9 | 71.4 |
| 100 | 0 | 0.5 | 480 | 0.5 | 84.7 | 75.0 |
| 100 | 0 | 0.5 | 500 | 0.5 | 87.5 | 71.4 |
| 100 | 0 | 0.75 | 120 | 0.25 | 6.9 | 3.6 |
| 100 | 0 | 0.75 | 140 | 0.25 | 6.9 | 7.1 |
| 100 | 0 | 0.75 | 160 | 0.25 | 8.3 | 14.3 |
| 100 | 0 | 0.75 | 180 | 0.25 | 8.3 | 7.1 |
| 100 | 0 | 0.75 | 200 | 0.25 | 6.9 | 14.3 |
| 100 | 0 | 0.75 | 220 | 0.25 | 9.7 | 14.3 |
| 100 | 0 | 0.75 | 240 | 0.25 | 12.5 | 21.4 |
| 100 | 0 | 0.75 | 260 | 0.25 | 19.4 | 17.9 |
| 100 | 0 | 0.75 | 280 | 0.25 | 20.8 | 21.4 |
| 100 | 0 | 0.75 | 300 | 0.25 | 26.4 | 17.9 |
| 100 | 0 | 0.75 | 320 | 0.25 | 27.8 | 14.3 |
| 100 | 0 | 0.75 | 340 | 0.25 | 34.7 | 21.4 |
| 100 | 0 | 0.75 | 360 | 0.25 | 33.3 | 32.1 |
| 100 | 0 | 0.75 | 380 | 0.25 | 27.8 | 17.9 |
| 100 | 0 | 0.75 | 400 | 0.25 | 44.4 | 42.9 |
| 100 | 0 | 0.75 | 420 | 0.25 | 44.4 | 39.3 |
| 100 | 0 | 0.75 | 440 | 0.25 | 41.7 | 53.6 |
| 100 | 0 | 0.75 | 460 | 0.25 | 41.7 | 46.4 |
| 100 | 0 | 0.75 | 480 | 0.25 | 43.1 | 46.4 |
| 100 | 0 | 0.75 | 500 | 0.25 | 45.8 | 46.4 |

Table S4. Distribution of risky choices across all lottery options in the sweet-liking and sweet-disliking groups in the lottery choice task, Experiment 2.

| **Delay of the sooner option** | **Delay of the delayed option** | **Sooner reward** | **Delayed reward** | **Percent of delayed choices across sweet-dislikers** | **Percent of delayed choices across sweet-likers** |
| --- | --- | --- | --- | --- | --- |
| 0 | 2 | 50 | 100 | 87.50 | 96.43 |
| 0 | 2 | 50 | 150 | 94.44 | 96.43 |
| 0 | 2 | 50 | 200 | 97.22 | 100.00 |
| 0 | 2 | 50 | 250 | 98.61 | 100.00 |
| 0 | 2 | 50 | 300 | 97.22 | 96.43 |
| 0 | 2 | 50 | 350 | 95.83 | 100.00 |
| 0 | 2 | 50 | 400 | 93.06 | 100.00 |
| 0 | 2 | 50 | 450 | 98.61 | 96.43 |
| 0 | 2 | 50 | 500 | 95.83 | 100.00 |
| 0 | 2 | 100 | 150 | 87.50 | 96.43 |
| 0 | 2 | 100 | 200 | 91.67 | 96.43 |
| 0 | 2 | 100 | 250 | 97.22 | 96.43 |
| 0 | 2 | 100 | 300 | 95.83 | 96.43 |
| 0 | 2 | 100 | 350 | 95.83 | 96.43 |
| 0 | 2 | 100 | 400 | 97.22 | 100.00 |
| 0 | 2 | 100 | 450 | 97.22 | 96.43 |
| 0 | 2 | 100 | 500 | 97.22 | 100.00 |
| 0 | 2 | 150 | 200 | 86.11 | 85.71 |
| 0 | 2 | 150 | 250 | 91.67 | 92.86 |
| 0 | 2 | 150 | 300 | 94.44 | 100.00 |
| 0 | 2 | 150 | 350 | 94.44 | 100.00 |
| 0 | 2 | 150 | 400 | 98.61 | 100.00 |
| 0 | 2 | 150 | 450 | 98.61 | 96.43 |
| 0 | 2 | 150 | 500 | 97.22 | 100.00 |
| 0 | 14 | 50 | 100 | 69.44 | 67.86 |
| 0 | 14 | 50 | 150 | 86.11 | 92.86 |
| 0 | 14 | 50 | 200 | 90.28 | 96.43 |
| 0 | 14 | 50 | 250 | 94.44 | 96.43 |
| 0 | 14 | 50 | 300 | 94.44 | 96.43 |
| 0 | 14 | 50 | 350 | 97.22 | 96.43 |
| 0 | 14 | 50 | 400 | 97.22 | 92.86 |
| 0 | 14 | 50 | 450 | 97.22 | 96.43 |
| 0 | 14 | 50 | 500 | 98.61 | 100.00 |
| 0 | 14 | 100 | 150 | 61.11 | 67.86 |
| 0 | 14 | 100 | 200 | 77.78 | 92.86 |
| 0 | 14 | 100 | 250 | 84.72 | 85.71 |
| 0 | 14 | 100 | 300 | 91.67 | 100.00 |
| 0 | 14 | 100 | 350 | 93.06 | 92.86 |
| 0 | 14 | 100 | 400 | 95.83 | 96.43 |
| 0 | 14 | 100 | 450 | 94.44 | 92.86 |
| 0 | 14 | 100 | 500 | 95.83 | 96.43 |
| 0 | 14 | 150 | 200 | 51.39 | 67.86 |
| 0 | 14 | 150 | 250 | 73.61 | 82.14 |
| 0 | 14 | 150 | 300 | 87.50 | 85.71 |
| 0 | 14 | 150 | 350 | 90.28 | 92.86 |
| 0 | 14 | 150 | 400 | 94.44 | 100.00 |
| 0 | 14 | 150 | 450 | 97.22 | 96.43 |
| 0 | 14 | 150 | 500 | 97.22 | 100.00 |
| 0 | 30 | 50 | 100 | 48.61 | 53.57 |
| 0 | 30 | 50 | 150 | 77.78 | 82.14 |
| 0 | 30 | 50 | 200 | 83.33 | 85.71 |
| 0 | 30 | 50 | 250 | 90.28 | 85.71 |
| 0 | 30 | 50 | 300 | 91.67 | 96.43 |
| 0 | 30 | 50 | 350 | 98.61 | 96.43 |
| 0 | 30 | 50 | 400 | 95.83 | 96.43 |
| 0 | 30 | 50 | 450 | 97.22 | 100.00 |
| 0 | 30 | 50 | 500 | 94.44 | 100.00 |
| 0 | 30 | 100 | 150 | 37.50 | 53.57 |
| 0 | 30 | 100 | 200 | 56.94 | 71.43 |
| 0 | 30 | 100 | 250 | 69.44 | 67.86 |
| 0 | 30 | 100 | 300 | 86.11 | 78.57 |
| 0 | 30 | 100 | 350 | 86.11 | 89.29 |
| 0 | 30 | 100 | 400 | 88.89 | 92.86 |
| 0 | 30 | 100 | 450 | 90.28 | 92.86 |
| 0 | 30 | 100 | 500 | 94.44 | 92.86 |
| 0 | 30 | 150 | 200 | 43.06 | 64.29 |
| 0 | 30 | 150 | 250 | 59.72 | 64.29 |
| 0 | 30 | 150 | 300 | 66.67 | 75.00 |
| 0 | 30 | 150 | 350 | 83.33 | 75.00 |
| 0 | 30 | 150 | 400 | 86.11 | 92.86 |
| 0 | 30 | 150 | 450 | 93.06 | 85.71 |
| 0 | 30 | 150 | 500 | 91.67 | 92.86 |
| 0 | 60 | 50 | 100 | 36.11 | 53.57 |
| 0 | 60 | 50 | 150 | 62.50 | 64.29 |
| 0 | 60 | 50 | 200 | 76.39 | 82.14 |
| 0 | 60 | 50 | 250 | 73.61 | 75.00 |
| 0 | 60 | 50 | 300 | 83.33 | 85.71 |
| 0 | 60 | 50 | 350 | 84.72 | 89.29 |
| 0 | 60 | 50 | 400 | 90.28 | 89.29 |
| 0 | 60 | 50 | 450 | 90.28 | 100.00 |
| 0 | 60 | 50 | 500 | 91.67 | 92.86 |
| 0 | 60 | 100 | 150 | 29.17 | 42.86 |
| 0 | 60 | 100 | 200 | 37.50 | 64.29 |
| 0 | 60 | 100 | 250 | 55.56 | 67.86 |
| 0 | 60 | 100 | 300 | 72.22 | 82.14 |
| 0 | 60 | 100 | 350 | 70.83 | 71.43 |
| 0 | 60 | 100 | 400 | 80.56 | 89.29 |
| 0 | 60 | 100 | 450 | 88.89 | 92.86 |
| 0 | 60 | 100 | 500 | 88.89 | 92.86 |
| 0 | 60 | 150 | 200 | 33.33 | 57.14 |
| 0 | 60 | 150 | 250 | 43.06 | 64.29 |
| 0 | 60 | 150 | 300 | 59.72 | 67.86 |
| 0 | 60 | 150 | 350 | 65.28 | 67.86 |
| 0 | 60 | 150 | 400 | 76.39 | 75.00 |
| 0 | 60 | 150 | 450 | 84.72 | 78.57 |
| 0 | 60 | 150 | 500 | 90.28 | 85.71 |
| 0 | 90 | 50 | 100 | 33.33 | 42.86 |
| 0 | 90 | 50 | 150 | 58.33 | 53.57 |
| 0 | 90 | 50 | 200 | 61.11 | 71.43 |
| 0 | 90 | 50 | 250 | 68.06 | 64.29 |
| 0 | 90 | 50 | 300 | 80.56 | 89.29 |
| 0 | 90 | 50 | 350 | 81.94 | 82.14 |
| 0 | 90 | 50 | 400 | 81.94 | 85.71 |
| 0 | 90 | 50 | 450 | 90.28 | 89.29 |
| 0 | 90 | 50 | 500 | 88.89 | 92.86 |
| 0 | 90 | 100 | 150 | 27.78 | 42.86 |
| 0 | 90 | 100 | 200 | 36.11 | 57.14 |
| 0 | 90 | 100 | 250 | 44.44 | 60.71 |
| 0 | 90 | 100 | 300 | 59.72 | 71.43 |
| 0 | 90 | 100 | 350 | 69.44 | 82.14 |
| 0 | 90 | 100 | 400 | 84.72 | 82.14 |
| 0 | 90 | 100 | 450 | 81.94 | 89.29 |
| 0 | 90 | 100 | 500 | 87.50 | 89.29 |
| 0 | 90 | 150 | 200 | 36.11 | 46.43 |
| 0 | 90 | 150 | 250 | 41.67 | 60.71 |
| 0 | 90 | 150 | 300 | 50.00 | 64.29 |
| 0 | 90 | 150 | 350 | 63.89 | 71.43 |
| 0 | 90 | 150 | 400 | 70.83 | 75.00 |
| 0 | 90 | 150 | 450 | 69.44 | 71.43 |
| 0 | 90 | 150 | 500 | 81.94 | 82.14 |
| 2 | 14 | 100 | 450 | 93.06 | 100.00 |
| 2 | 14 | 150 | 500 | 94.44 | 100.00 |
| 30 | 60 | 50 | 100 | 52.78 | 75.00 |
| 30 | 60 | 50 | 150 | 84.72 | 85.71 |
| 30 | 60 | 50 | 200 | 84.72 | 100.00 |
| 30 | 60 | 50 | 250 | 88.89 | 100.00 |
| 30 | 60 | 50 | 300 | 93.06 | 100.00 |
| 30 | 60 | 50 | 350 | 91.67 | 100.00 |
| 30 | 60 | 50 | 400 | 93.06 | 100.00 |
| 30 | 60 | 50 | 450 | 93.06 | 100.00 |
| 30 | 60 | 50 | 500 | 91.67 | 100.00 |
| 30 | 60 | 100 | 150 | 31.94 | 57.14 |
| 30 | 60 | 100 | 200 | 68.06 | 75.00 |
| 30 | 60 | 100 | 250 | 76.39 | 82.14 |
| 30 | 60 | 100 | 300 | 86.11 | 96.43 |
| 30 | 60 | 100 | 350 | 87.50 | 96.43 |
| 30 | 60 | 100 | 400 | 93.06 | 96.43 |
| 30 | 60 | 100 | 450 | 90.28 | 100.00 |
| 30 | 60 | 100 | 500 | 93.06 | 100.00 |
| 30 | 60 | 150 | 200 | 37.50 | 50.00 |
| 30 | 60 | 150 | 250 | 63.89 | 67.86 |
| 30 | 60 | 150 | 300 | 81.94 | 78.57 |
| 30 | 60 | 150 | 350 | 80.56 | 92.86 |
| 30 | 60 | 150 | 400 | 91.67 | 92.86 |
| 30 | 60 | 150 | 450 | 94.44 | 92.86 |
| 30 | 60 | 150 | 500 | 91.67 | 100.00 |
| 30 | 90 | 50 | 100 | 51.39 | 67.86 |
| 30 | 90 | 50 | 150 | 68.06 | 71.43 |
| 30 | 90 | 50 | 200 | 77.78 | 89.29 |
| 30 | 90 | 50 | 250 | 86.11 | 89.29 |
| 30 | 90 | 50 | 300 | 86.11 | 89.29 |
| 30 | 90 | 50 | 350 | 88.89 | 96.43 |
| 30 | 90 | 50 | 400 | 90.28 | 100.00 |
| 30 | 90 | 50 | 450 | 94.44 | 96.43 |
| 30 | 90 | 50 | 500 | 91.67 | 96.43 |
| 30 | 90 | 100 | 150 | 31.94 | 57.14 |
| 30 | 90 | 100 | 200 | 38.89 | 57.14 |
| 30 | 90 | 100 | 250 | 61.11 | 71.43 |
| 30 | 90 | 100 | 300 | 72.22 | 71.43 |
| 30 | 90 | 100 | 350 | 77.78 | 82.14 |
| 30 | 90 | 100 | 400 | 88.89 | 89.29 |
| 30 | 90 | 100 | 450 | 88.89 | 96.43 |
| 30 | 90 | 100 | 500 | 94.44 | 96.43 |
| 30 | 90 | 150 | 200 | 29.17 | 42.86 |
| 30 | 90 | 150 | 250 | 41.67 | 60.71 |
| 30 | 90 | 150 | 300 | 62.50 | 71.43 |
| 30 | 90 | 150 | 350 | 68.06 | 64.29 |
| 30 | 90 | 150 | 400 | 76.39 | 82.14 |
| 30 | 90 | 150 | 450 | 79.17 | 89.29 |
| 30 | 90 | 150 | 500 | 87.50 | 96.43 |

Table S5. Distribution of delayed choices across all delay discounting options in the sweet-liking and sweet-disliking groups in the delay discounting task, Experiment 2.


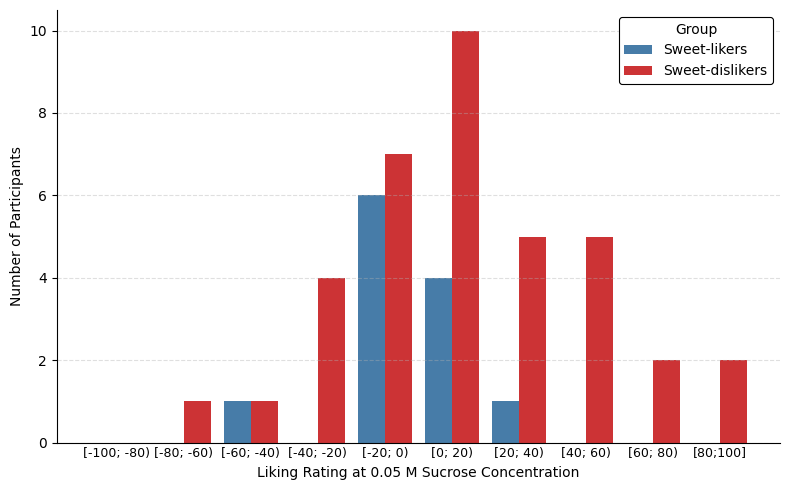


Figure S1. Distribution of sweet-liking ratings at the 0.05 M sucrose concentration in the sweet-liking and sweet-disliking groups, Experiment 1.


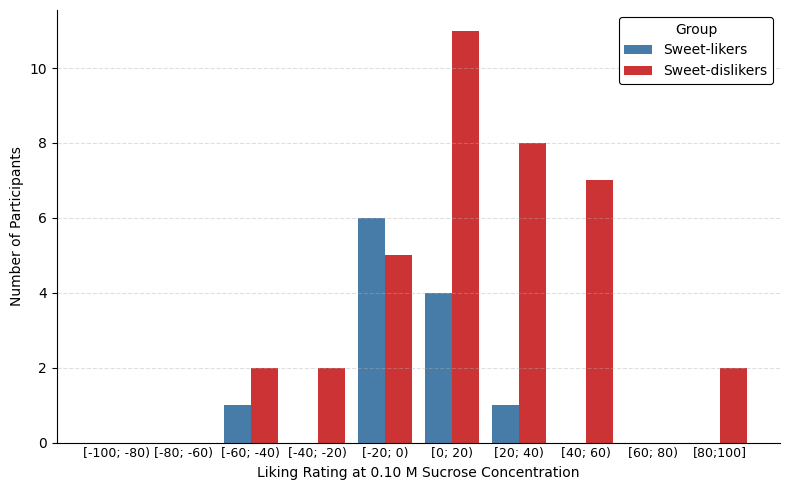


Figure S2. Distribution of sweet-liking ratings at the 0.10 M sucrose concentration in the sweet-liking and sweet-disliking groups, Experiment 1.


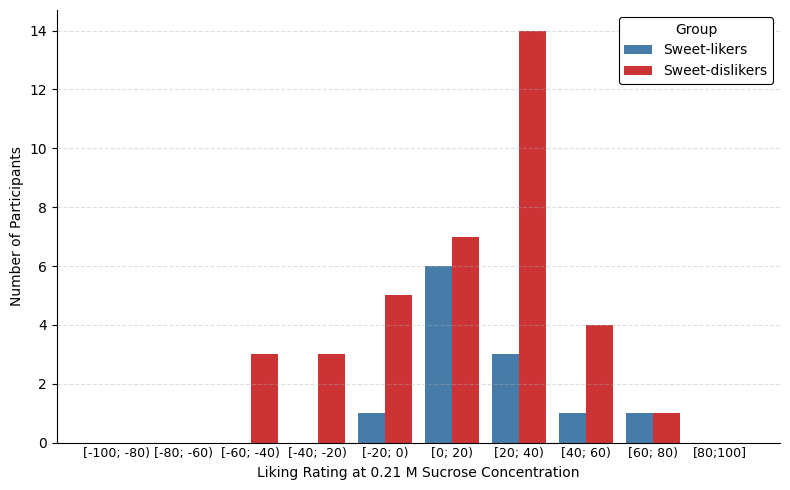


Figure S3. Distribution of sweet-liking ratings at the 0.21 M sucrose concentration in the sweet-liking and sweet-disliking groups, Experiment 1.


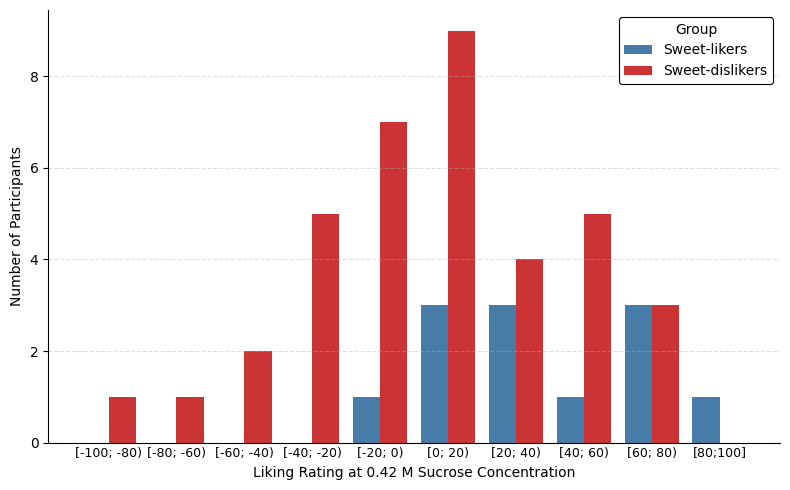


Figure S4. Distribution of sweet-liking ratings at the 0.42 M sucrose concentration in the sweet-liking and sweet-disliking groups, Experiment 1.


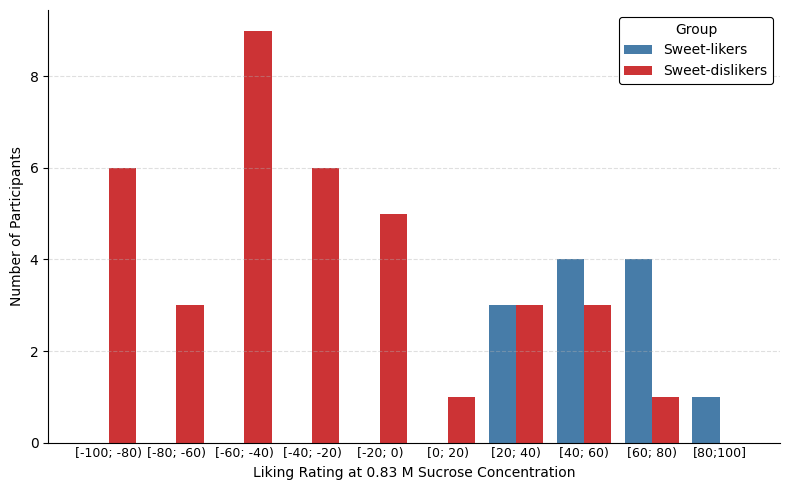


Figure S5. Distribution of sweet-liking ratings at the 0.83 M sucrose concentration in the sweet-liking and sweet-disliking groups, Experiment 1.


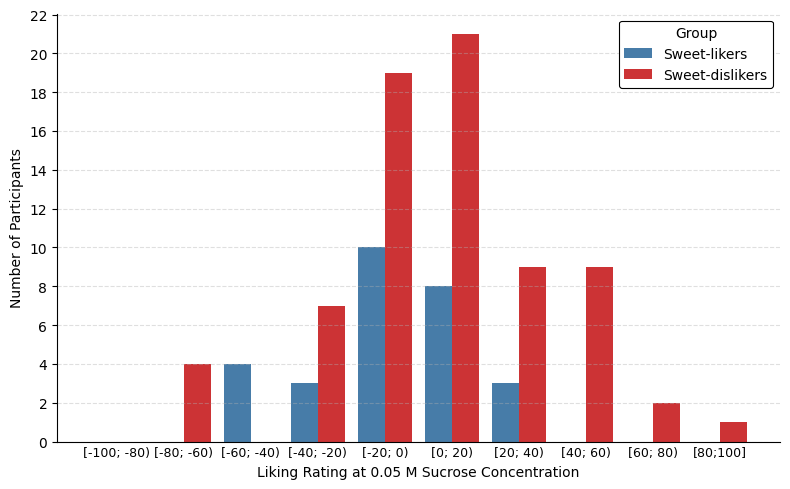


Figure S6. Distribution of sweet-liking ratings at the 0.05 M sucrose concentration in the sweet-liking and sweet-disliking groups, Experiment 2.


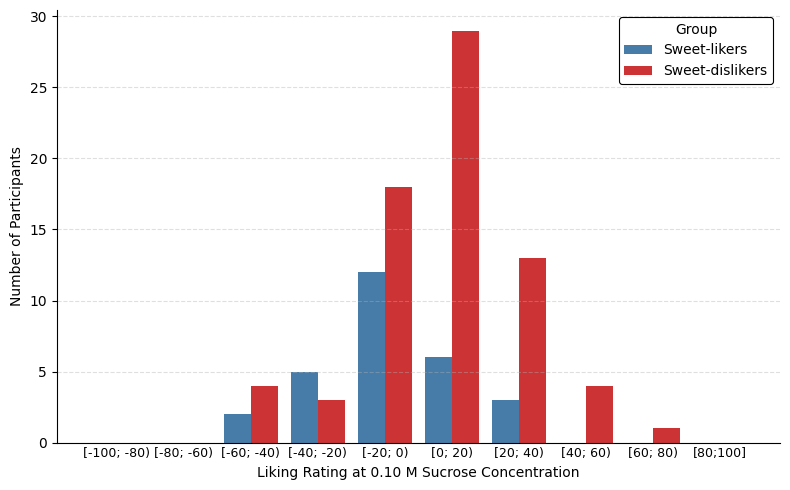


Figure S7. Distribution of sweet-liking ratings at the 0.10 M sucrose concentration in the sweet-liking and sweet-disliking groups, Experiment 2.


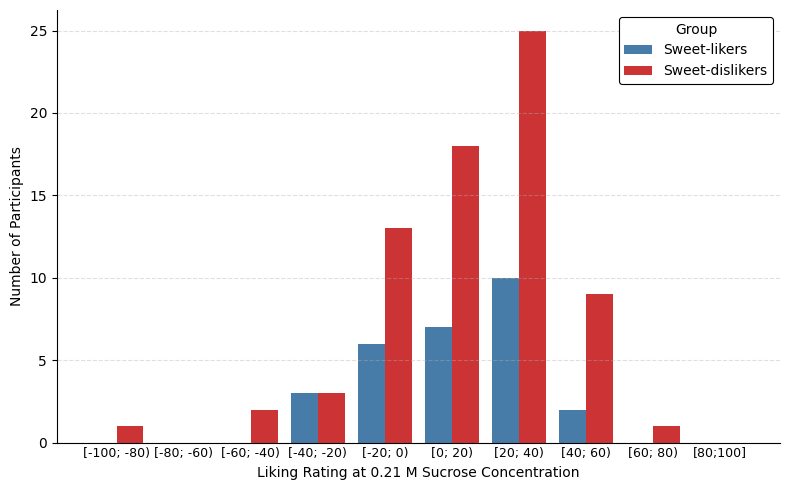


Figure S8. Distribution of sweet-liking ratings at the 0.21 M sucrose concentration in the sweet-liking and sweet-disliking groups, Experiment 2.


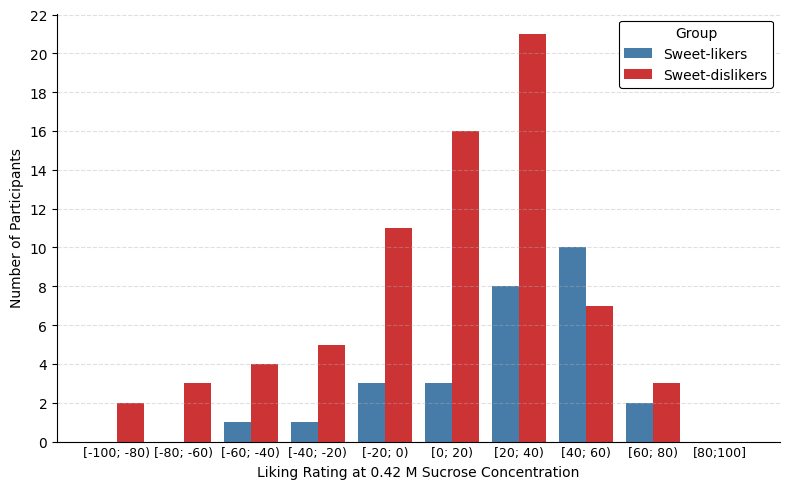


Figure S9. Distribution of sweet-liking ratings at the 0.42 M sucrose concentration in the sweet-liking and sweet-disliking groups, Experiment 2.


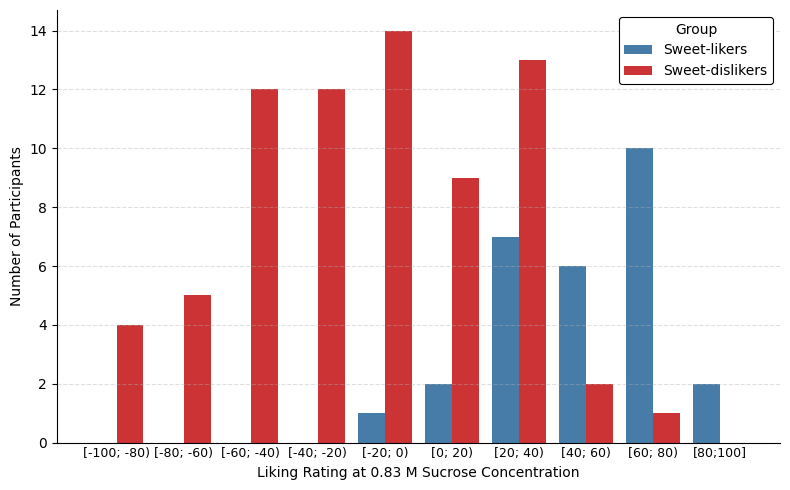


Figure S10. Distribution of sweet-liking ratings at the 0.83 M sucrose concentration in the sweet-liking and sweet-disliking groups, Experiment 2.
